# The spatiotemporal dynamics of microglia across the human lifespan

**DOI:** 10.1016/j.devcel.2022.07.015

**Published:** 2022-09-12

**Authors:** David A. Menassa, Tim A.O. Muntslag, Maria Martin-Estebané, Liam Barry-Carroll, Mark A. Chapman, Istvan Adorjan, Teadora Tyler, Bethany Turnbull, Matthew J.J. Rose-Zerilli, James A.R. Nicoll, Zeljka Krsnik, Ivica Kostovic, Diego Gomez-Nicola

**Affiliations:** 1 School of Biological Sciences, University of Southampton, Southampton, United Kingdom; 2 Institute of Basic Medical Sciences, University of Oslo, Oslo, Norway; 3 Department of Anatomy, Histology and Embryology, Semmelweis University, Budapest, Hungary; 4 Cancer Sciences, Faculty of Medicine, University of Southampton, Southampton, United Kingdom; 5 Clinical and Experimental Sciences, Faculty of Medicine, University of Southampton, Southampton, United Kingdom; 6 Croatian Institute for Brain Research, University of Zagreb Medical School, Zagreb, Croatia

**Keywords:** neurodevelopment, proliferation, apoptosis, RNA-seq, single-cell RNA-seq

## Abstract

Microglia, the brain’s resident macrophages, shape neural development and are key neuroimmune hubs in the pathological signatures of neurodevelopmental disorders. Despite the importance of microglia, their development has not been carefully examined in the human brain, and most of our knowledge derives from rodents. We aimed to address this gap in knowledge by establishing an extensive collection of 97 post-mortem tissues in order to enable quantitative, sex-matched, detailed analysis of microglia across the human lifespan. We identify the dynamics of these cells in the human telencephalon, describing waves in microglial density across gestation, infancy, and childhood, controlled by a balance of proliferation and apoptosis, which track key neurodevelopmental milestones. These profound changes in microglia are also observed in bulk RNA-seq and single-cell RNA-seq datasets. This study provides a detailed insight into the spatiotemporal dynamics of microglia across the human lifespan and serves as a foundation for elucidating how microglia contribute to shaping neurodevelopment in humans.

## Highlights

• A roadmap of microglial dynamics across the human lifespan
• The density of microglial cells follows a wave-like pattern during development
• Microglial fluctuations are spatiotemporally linked to key neurodevelopmental hallmarks

## Graphical abstract

**Figure:**
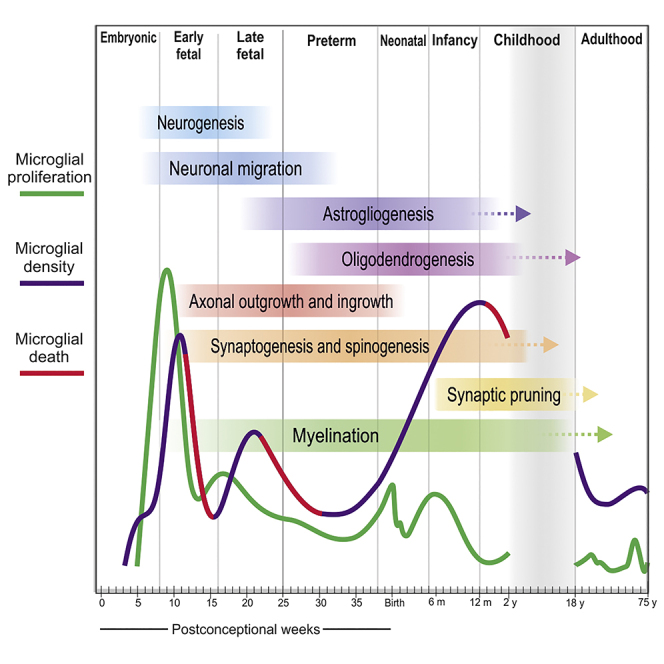

## Teaser

Microglial cells are pivotal players in brain development and function. However, most of our knowledge about their development derives from rodents. Menassa et al. describe the dynamics of microglia across the human lifespan from early gestation until old age, identifying distinct dynamics that are intimately associated with key neurodevelopmental hallmarks.

## Introduction

Microglia are the main resident immune cells of the brain. During development, their roles include the phagocytosis of neuronal precursors to restrict progenitor pool size (Cunningham et al., 2013), the modulation of forebrain wiring by guiding interneuron positioning (Squarzoni et al., 2014), the pruning of synapses (Paolicelli et al., 2011), and the formation and refinement of axonal tracts (Verney et al., 2010; Pont-Lezica et al., 2014; Squarzoni et al., 2014). In the adult, microglia retain key homeostatic functions, suppressing interneuron activation and modifying animal behavior (Badimon et al., 2020), regulating hippocampal neurogenesis (Sierra et al., 2010), and driving inflammation in a disease context (Gomez-Nicola and Perry, 2015).

However, most of our knowledge of microglial developmental dynamics is derived from rodent studies. In the mouse, microglia originate from erythromyeloid progenitors in the extraembryonic yolk sac (YS) from E7.5 (Ginhoux et al., 2010). These progenitors begin populating the brain primordium at E9.5 and their phenotypic specification into microglia is defined by intrinsic and brain-specific transcriptional regulators (Lavin et al., 2014; Gosselin et al., 2014; Bennett et al., 2018). The entire microglial population is generated by expansion during embryonic life and the early postnatal developmental stages, followed by a transient postnatal selection phase (Askew et al., 2017; Réu et al., 2017). Under homeostatic conditions, the population is maintained throughout life by cycles of slow self-renewal, estimated at approximately 0.69% per day (Askew et al., 2017).

Human microglial development has some similarities to mouse, as macrophages are detectable by 2–3 postconceptional weeks (pcw) in the blood islands of the extraembryonic YS (Nogales, 1993; Janossy et al., 1986; Popescu et al., 2019; Park et al., 2020), to later appear in the forebrain from the 3^rd^ pcw (Verney et al., 2010; Menassa and Gomez-Nicola, 2018). A series of descriptive post-mortem studies collectively report on microglial proliferation in clusters appearing in the ventral telencephalon and diencephalon from the 4^th^ pcw and continuing throughout fetal development (Monier et al., 2007; Monier et al., 2006; Verney et al., 2010). Recent single-cell transcriptomic studies suggest that microglial ontogenic pathways are conserved between human and mouse during embryonic development (Bian et al., 2020), with microglia progressing from an undifferentiated state toward a mature adult-like immunocompetent state from 11 pcw in humans (Kracht et al., 2020). The early arrival of microglia to the brain precedes the onset of pivotal processes of human cortical development, such as neurogenesis, neuronal migration, and gliogenesis (Menassa and Gomez-Nicola, 2018). In the adult, the population self-renews at a slow daily turnover rate, which has been estimated at 0.08%–2% (Askew et al., 2017; Réu et al., 2017). However, we lack a deep understanding of microglial spatiotemporal dynamics during human development, compared with the mouse, largely due to the scarcity of developing tissue available for research.

**Figure 1:**
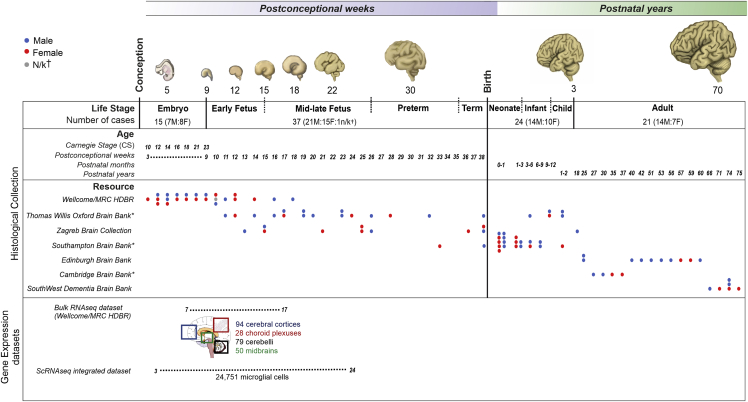
Overview of the study Post-mortem collection of human tissues across the lifespan with 52 prenatal and 45 postnatal cases, which adds up to n = 97. The overall number of cases collected, sampled, and analyzed was in excess of 130 (see STAR Methods section and Table S2). Temporal windows mapped onto key human neurodevelopmental milestones across the lifespan were defined consistent with existing classifications (Carroll et al., 2021; Silbereis et al., 2016; Kostović et al., 2002): the embryonic period (3–8 pcw), the early fetal period (9–15 pcw), the mid-late fetal period (16–25 pcw), the preterm period (26–35 pcw), the term period (36 pcw–birth), the neonatal period (0–1 month), the infancy period (1–12 months), childhood (1–3 years), and adulthood (>18 years). Gene expression datasets: bulk RNA-seq of 251 samples from the Wellcome MRC/HDBR resource in 4 anatomical regions between 7 and 17 postconceptional weeks and an integrated dataset of 24,751 microglial cells from 4 single-cell RNA-seq studies (Cao et al., 2020; Kracht et al., 2020; Bian et al., 2020; Fan et al., 2020) between 3 and 24 postconceptional weeks. F, female; M, male; MRC/HDBR, Medical Research Council/Human Developmental Biology Resource; † indicates not known, and ^∗^^indicates^ brain banks that are part of the BRAINUK network. Embryonic brain drawings were based on our own samples, and other model brains were redrawn and colored based on illustrations from Haymaker and Adams (1982).

We established an unprecedented collection of 97 post-mortem tissues (Figure 1), enabling quantitative, sex-matched, and detailed analysis of microglial dynamics across the human lifespan (3^rd^ pcw—75 years), in relation to their immunocompetent and neurogenetic roles. We identify developmental and postnatal waves of expansion and refinement, where the population undergoes marked changes in numbers, and we determine the contributions of proliferation, apoptosis, and migration to this process. We validate our identified critical windows in further analyses of datasets from 251 bulk RNA-seq samples and four single-cell RNA-seq (scRNA-seq) studies comprising 24,751 microglial cells spanning the embryonic and fetal ages (3–24 pcw). This study is pivotal for our understanding of human microglial biology, providing a granular view of the population dynamics across the life course and its intertwined nature with key neurodevelopmental processes. These findings serve as a solid basis for elucidating how microglia shape brain development in humans, key for understanding neurodevelopmental disorders.

## Results

### Brain colonization by microglia begins in the 4^th^ postconceptional week (CS12)

**Figure 2:**
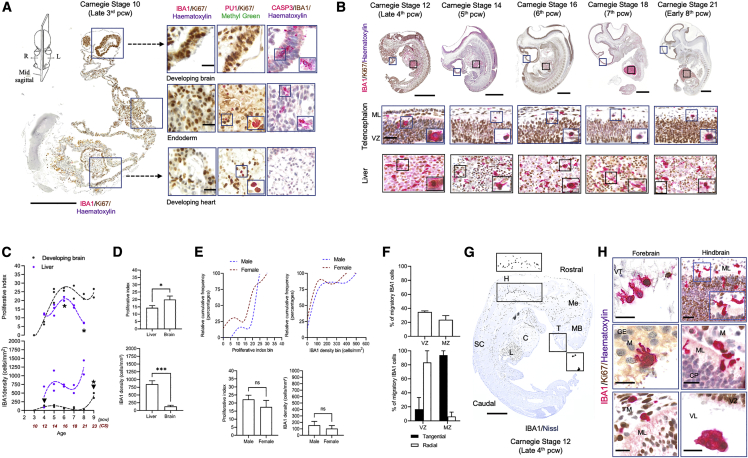
Early colonization of the human embryo (A) Representative sagittal cross-section through a CS10 (late 3^rd^ pcw) human embryo. Immunolabeling was done on consecutive 8-μm sections identifying the presence of PU.1 cells in organs apart from the brain and the absence of IBA1^+^ cells overall. Scale bars: 500 μm (left); 40 μm (right). (B) Representative sagittal sections through whole human embryos from CS12, the age of appearance of IBA1^+^ cells across the entire embryo, through to CS21. Insets show the location of proliferating IBA1^+^ cells in both the liver and the dorsal telencephalon. Scale bars: 1 mm (top); 100 μm (bottom). (C) IBA1 proliferative dynamics and cell densities in the developing brain (n = 14) and the liver (n = 11). Black arrows represent the two most relevant time points for microglial densities: CS12 (4^th^ pcw) for the first colonization of the brain rudiment and CS23 (9^th^ pcw), the transition from embryonic to early fetal life. (D) Mean IBA1 proliferation in the liver (n = 11) and the developing brain (n = 11) across the embryonic period. All data are shown as mean ± SEM. (E) Cumulative frequency distribution plots of densities and proliferative indices by sex in the developing brain (7F:7M) (top panel); mean differences between sexes in microglial densities and proliferative indices across the embryonic age in the developing brain (7F:7M) (bottom panel, data are shown as mean ± SEM). (F) Representative migratory profiles of IBA1^+^ cells in the bilaminar telencephalon at 5 pcw (n = 2). Data are shown as mean ± SEM. (G) IBA1^+^ cell distributions in the CS12 embryo showing very few cells in the forebrain and a much higher density of cells in the hindbrain and midbrain. Scale bars: 500 μm. (H) Entry routes of IBA1^+^ cells into the brain rudiment in the forebrain and the hindbrain. Scale bars: 100 μm. C, cardiac muscle; CP, cortical plate; GE, ganglionic eminence; H, hindbrain; L, liver; M, meninges; MB, midbrain; Me, mesenchyme; ML, mantle layer; SC, spinal cord; T, telencephalon; VZ, ventricular zone. For all panels, asterisks represent adjusted p value significance as follows: *^∗^*p < 0.05, ^∗∗^p < 0.001, ^∗∗∗^p < 0.0001, and ns p > 0.05.

The onset of embryonic circulation is concomitant with the development of the cardiovascular system, which in humans becomes functional from the late 3^rd^/early 4^th^ pcw (somites: 4–12; CS10 onward) (Tavian and Péault, 2005; O’Rahilly and Müller, 2010). At CS10, we identify early colonization of embryonic organs such as the heart by cells committed to the myeloid lineage (PU.1^+^; Figure 2A), with the expression of IBA1 not yet detected (Figure 2A). By CS12 (the end of the 4^th^ pcw), organ colonization by IBA1^+^ cells (Figure 2B) is profuse. Proliferation is highest at CS16 (6^th^ pcw) and lowest at CS21 (8^th^ pcw) in the liver (Kruskal-Wallis p < 0.05, multiple comparisons’ adjusted p values < 0.01) (Figure 2C). Liver macrophage densities are not significantly different between pcw across the embryonic age (Kruskal-Wallis p > 0.05, ns) (Figure 2C).

At CS10, no IBA1^+^ or PU.1^+^ myeloid cells are detected in the brain rudiment (Figure 2A). By CS12, the first wave of brain colonization begins, with IBA1^+^ macrophages seeding the forebrain, midbrain, and hindbrain (Figure 2B). Microglial proliferation increases sharply from CS12 to CS14 and remains relatively stable across the embryonic age with no significant differences in mean proliferation levels between pcw (Kruskal-Wallis test, p > 0.05, ns) (Figure 2C). The brain’s proliferative index is much higher than the liver’s (Mann-Whitney U test, p < 0.05) but its IBA1 densities are significantly lower (Mann-Whitney U test, p < 0.001) (Figure 2D). The first detectable expansion phase of the population is observed after a sharp increase in IBA1^+^ cell density at CS23 (9^th^ pcw) compared with every pre-CS23 pcw (Kruskal-Wallis test, p < 0.05, multiple comparisons’ adjusted post hoc p values < 0.01) (Figure 2C).

In the developing brain, we found no significant differences between the cumulative distributions of microglia by sex for both density and proliferation (2-sample Kolmogorov-Smirnov test, p > 0.05, ns) (Figure 2E, top). When we considered the mean proliferation and density by sex across the embryonic age, we also found no differences between males and females (Mann-Whitney U test, p > 0.05) (Figure 2E, bottom). Early in development (5^th^ pcw), migratory microglia (IBA1^+^) account for ∼40% of the total population in the dorsal telencephalon, which at this age is bilaminar with a proliferative zone, the ventricular zone (VZ), and a plexiform mantle layer (ML) (Figures 2B and 2F, top). Based on a morphometric analysis, most of the migration in the VZ appears radial, indicating the colonization of adjacent layers, whereas microglia in the ML are characterized by a tangential migratory phenotype, suggesting expansion within a layer (Figure 2F, bottom). Morphologically, microglia in the bilaminar telencephalon are amoeboid, with or without a proliferative core, or migratory (tangential or radial) (Figure S1A).

Microglial densities and proliferation vary between regions: cell densities tend to be highest in the hindbrain at CS12, compared with the forebrain and midbrain (Figures 2G and S1B), through to CS21, and proliferation is significantly higher in the midbrain compared with the hindbrain and the forebrain (Friedman’s test, p < 0.05) (Figure S1B).

In sum, we can date the arrival of microglial progenitors to the brain at CS12 (4^th^ pcw), with the population rapidly engaging in proliferative and migratory activities.

### The density of the microglial population fluctuates by waves of proliferation followed by cell death in the developing cortex

The future neocortex is formed from the dorsal telencephalon in the forebrain, which is where we focused all our subsequent analyses. Between CS12 (4^th^ pcw) and CS21 (8^th^ pcw), microglial proliferation is restricted to the ML (Figure 2B) and cells are seen entering the brain parenchyma from the VZ, the meningeal compartment, and the ganglionic eminence (GE) (Figure 2H).

The last stage of embryonic life is CS23 (9^th^ pcw) (O’Rahilly and Müller, 2010), equivalent to the late 8^th^ or early 9^th^ pcw, followed by fetal life. We observed key proliferation waves characterizing the expansion of the microglial population during embryonic and early fetal lives.

**Figure 3:**
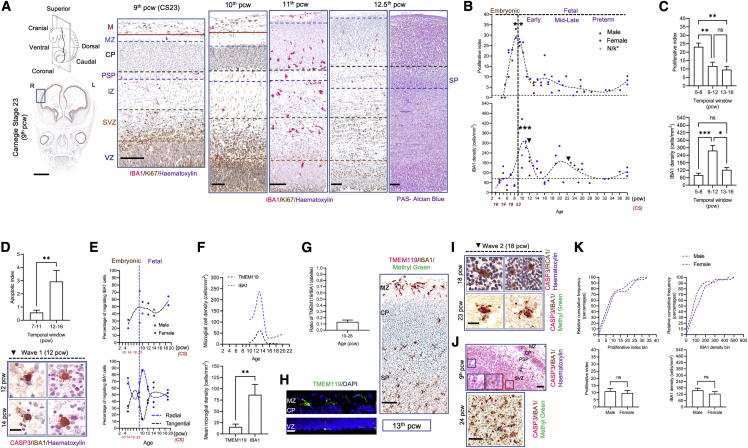
Developmental dynamics of microglia in the cortex (A) Representative laminar structure of the developing cortex with its transient zones observed from CS23 (9^th^ pcw) in humans and representative cortical columns from 10 to 12 pcw showing (1) the development of the pre-subplate to the subplate at 12.5 pcw and the alignment of microglial cells at the CP-PSP/SP boundary and (2) the distribution of microglial cells across transient zones. Scale bars: 2 mm (left); 100 μm (right). (B) Corrected microglial densities (against fold change in frontal telencephalic wall thickness with age) and proliferative dynamics in the telencephalon during development (CS10 [late 3^rd^/early 4^th^ pcw]) until term (38 pcw) (n = 50). ^∗^n/k, not known. Embryonic and early fetal temporal windows are most significant for proliferation against other temporal windows, while the early fetal window is the most significant against other temporal windows. (C) Equally spaced temporal windows for proliferation levels (top) and densities (bottom) around the most significant first wave. Data are represented as mean ± SEM. (D) Mean apoptotic index around the first peak of densities (n = 15, 8 cases between 7 and 11 pcw and 7 cases between 12 and 16 pcw) (top panel). Data are represented as mean ± SEM. Representative microglial cell death photomicrographs observed in wave 1 following the decrease in densities (bottom panel, black arrows in B). Scale bars: 20 μm. (E) Migratory profile of microglia and type of migration in representative cases from the telencephalon (n = 12). (F) Representative profile (top panel) of TMEM119^+^ and IBA1^+^ cell densities around the first significant density wave (10–16 pcw) (n = 6). Mean TMEM119^+^ and IBA1^+^ cell densities across the prenatal period (10–28 pcw, n = 10) (bottom panel). Data are shown as mean ± SEM. (G) Ratio of labeled TMEM119^+^/IBA1^+^ cells during the prenatal period (10–28 pcw, n = 10). Data are presented as mean ±SEM. (H) Representative confocal images of TMEM119^+^ cells in the MZ and the VZ of a 13-pcw case (left) and double labeling of TMEM119/IBA1 in bright field in a neocortical column at 13 pcw observed in the MZ (right). Scale bars (left): 50 μm; scale bars (right): 100 μm. (I) Wave 2 microglial cell death at 18 and 23 pcw. Scale bars: 20 μm. (J) Non-microglial death observed in the GE and in cortical transient zones at CS23 (9^th^ pcw) (top, scale bars: 100 μm) and at 24 pcw (bottom, scale bars: 50 μm). (K) Proliferative dynamics and densities by sex across human development shown as relative cumulative frequency distribution plots (top panel) and mean values between the sexes (27M:23F) (bottom panel). Data are shown as mean ± SEM. R, right; L, left; CP, cortical plate; GE, ganglionic eminence; IZ, intermediate zone; M, meninges; MZ, marginal zone; PSP, pre-subplate; SP, subplate; SVZ, subventricular zone; VZ, ventricular zone. For all panels, asterisks represent adjusted p value significance as follows: ^∗^p < 0.05, ^∗∗^p < 0.001, ^∗∗∗^p < 0.0001, and ns p > 0.05.

This first wave is the most significant and coincides with the appearance of multiple transient layers in the telencephalon, including the cortical plate (CP) and the pre-subplate (PSP), which becomes the subplate (SP) at the 12.5^th^ pcw (Figures 3A, 3B, and S1C). This first wave is at the transition between embryonic and fetal life (late 8^th^ to early 9^th^ pcw) (O’Rahilly and Müller, 2010; Kostović et al., 2002), as evaluated with an excess test for bimodal distributions (B = 100 replicas, modes: 2.04, 22.46; antimode: 16.35, p > 0.05 [Ameijeiras-Alonso et al., 2019]). Mean proliferation levels are highest in the embryonic window (5–8 pcw) compared with early fetal windows (9–12 pcw, 13–16 pcw) (Kruskal-Wallis test p < 0.01, multiple comparisons’ adjusted post hoc p values < 0.01) (Figure 3C).

This increase in microglial proliferation (IBA1^+^Ki67^+^) is followed by a peak in density, observed subsequently at 10–12 pcw (Figures 3B and 3C) in the frontal telencephalic wall. This first density wave in early fetal life is the most significant, evidenced by an excess test for bimodal distributions (B = 100 replicas, modes: 85.77, 484.16; antimode: 426.84; p > 0.05) (Figure 3B). Mean density levels are highest in the early fetal window (9–12 pcw) compared with 5–8 pcw and 13–16 pcw (Kruskal-Wallis test p < 0.001, multiple comparison adjusted post hoc p values < 0.001) (Figure 3C, bottom panel).

From 12 pcw, the microglial density drops to baseline levels (Figure 3B) due to microglial apoptosis that we detect by cleaved caspase-3 labeling in microglia between 12 and 16 pcw (Figure 3D). The mean percentage of apoptotic microglia before the density starts decreasing from 12 pcw was 0.58% ± 0.18% (7–11 pcw), increasing to 2.94% ± 0.83% after 12 pcw until 16 pcw (Mann-Whitney U test, p = 0.007) (Figure 3D).

Between 9 and 10 pcw we also detect a significant transient switch in the migratory phenotype of microglia, with most cells adopting a radial migratory pattern (Figure 3E) across all telencephalic layers (Figure S2), suggestive of a local colonization process being coupled to cycles of expansion.

We complemented the IBA1 quantifications with an analysis of TMEM119 expression. This study may pose an opportunity to assess TMEM119, as there are conflicting reports in the literature about its validity as a bona fide microglial marker (Bennett et al., 2016; Vankriekelsvenne et al., 2022). TMEM119 cell density changes tracked the pattern followed by IBA1^+^ cells between 9 and 16 pcw (Figure 3F). However, TMEM119 cell densities were significantly lower compared with IBA1 cells during this window (Wilcoxon test, p < 0.01) (Figure 3F). TMEM119 densities correlated significantly with IBA1 densities (Spearman’s r = 0.84, p < 0.005, Figure S3A) during development. The ratio of TMEM119/IBA1 labeled cells was ∼10% between 10 and 28 pcw (Figure 3G), with a colocalization degree of ∼90% (Figures 3H and S3B). Topographically, TMEM119^+^ cells were strongly detected in the MZ and the VZ (Figure 3H). Sporadic expression of TMEM119 was detected in the lower portion of the CP but not in any other middle layers, while IBA1 expression was consistent (Figure 3H). TMEM119^+^ cells were not proliferative (Figure S3C) and co-expressed lysosomal marker CD68 particularly in the MZ (Figure S3D).

The second wave of proliferation was less significant (bimodal distribution excess test p > 0.05) but will be described because patterns similar to the first wave were observed. The second proliferation window coincides with the early expansion of the human SP between 13 and 16 pcw (Kostović, 2020) (Figures 3A and 3B). The increase in proliferation was followed by an increase in density, which peaked at 20 pcw (Figure 3B). Thereafter, the density drops to baseline levels and closely matches the mean density values obtained in the adult (mean N_D_ = 84.63 ± 4.41 [mean cells/mm^2^ ± SEM]). This drop in density can be explained by microglial apoptosis (cleaved caspase-3^+^) between 18 and 24 pcw (Figure 3I). We also detect non-microglial apoptosis at various time points during development (Figure 3J).

The wave-like pattern followed by the microglial population during development is observed in both females and males (Figures 3B and 3K). No significant differences in microglial proliferation or density between the sexes were observed (Figure 3K) (Kolmogorov-Smirnov test, p > 0.05 for distribution plots between males and females [Figure 3K, top], and Mann-Whitney U test for mean proliferation and density comparisons between males and females, p > 0.05 [Figure 3K, bottom]).

**Figure 4:**
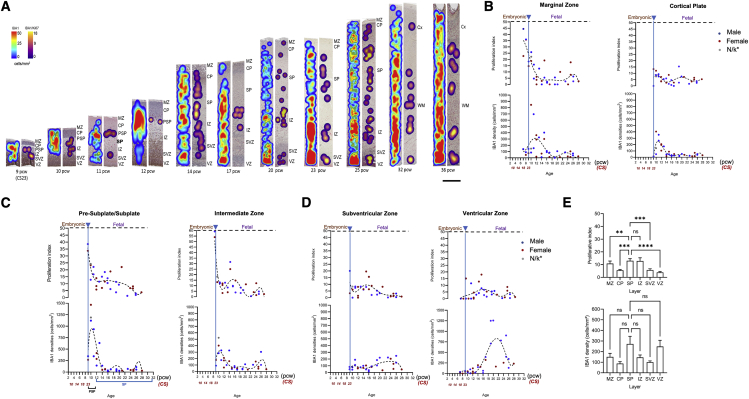
Spatiotemporal microglial dynamics within the developing cortex (A) Representative heatmaps showing microglial dynamics throughout development in the human cortex. Heatmaps were constructed based on the density of immunoreactive cells. Density of all IBA1 immunoreactive cells are represented by blue-red spectral heatmaps (left column of each stage), while IBA1^+^/Ki67^+^ double-positive cells are represented by purple-yellow spectral heatmaps (right column of each stage). Approximate densities are shown on the color bar with minimal density being blue/purple, maximum density being red/yellow. All cortical columns are 450-μm wide. (B–D) (B) Proliferation and densities of microglia in the marginal zone (n = 35), cortical plate (n = 31); (C) the subplate (n = 31) and the intermediate zone (n = 31); (D) the subventricular (n = 31) and the ventricular zones (n = 35). (E) Proliferation and densities in the subplate, the intermediate zone, and remaining cortical layers. Data are shown as mean ±SEM. CP, cortical plate; GM, gray matter; IZ, intermediate zone; MZ, marginal zone; PSP, pre-subplate; SP, subplate; SVZ, subventricular zone; VZ, ventricular zone; WM, white matter. For all panels, asterisks represent adjusted p value significance as follows: ^∗^p < 0.05, ^∗∗^p < 0.001, ^∗∗∗^p < 0.0001, and ns p > 0.05.

Microglial expansion is not uniform across transient zones of the developing cortex. As development progresses, we observed a pattern of “hot spots” of local proliferation preceding an increase in local density in subsequent time points, which is replicated across layers (Figures 4A–4D). Every layer has a different timing: whereas densities peak earlier in development for the MZ, CP, and SP (Figures 4A–4C), they do so much later for the subventricular zone (SVZ) and the VZ (Figures 4A and 4D). Densities and proliferation are significant in the SP and intermediate zone (IZ) (Figure 4E) (Kruskal-Wallis test, adjusted p < 0.01). Migration patterns vary within each layer (Figure S2), perhaps indicating movement of cells between adjacent layers.

During fetal life (9 pcw–term), microglial morphology is diverse: amoeboid, migratory (radial and tangential), clustered, ramified, bipolar, and phagocytic/activated with pouches in the vicinity of the cell (Figures S1A and S4C–S4F). Some morphologies can be linked to function, such as proliferative (IBA1^+^Ki67^+^ cells), apoptotic (IBA1^+^Casp3^+^), and homeostatic (TMEM119^+^) (Figures S1C and S2).

Altogether, post colonization at CS12 (4^th^ pcw), the microglial population follows a wave-like pattern of significant increases in density preceded by increases in proliferation, later refined by cell death, with the ultimate consequence of densities stabilizing at levels observed in the adult after every wave.

### Microglia undergo a wave-like pattern of transcriptional changes

**Figure 5:**
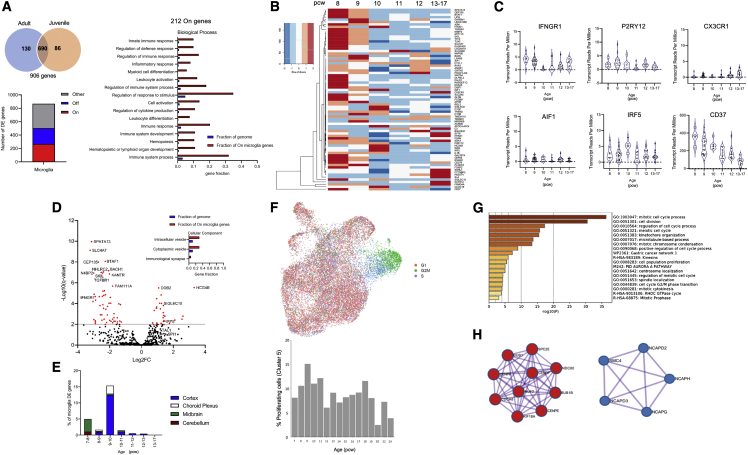
Microglial gene signature during development (A) 906 genes chosen from two published microglial-specific cortical adult and juvenile gene lists (Gosselin et al., 2017; Galatro et al., 2017). Bar plot shows the number of constitutively expressed microglial genes (“On”), unexpressed genes (“Off”) and those with sporadic expression in the developing cortex (“Other”) with gene ontology analysis of constitutively expressed genes only. (B) Heatmap of highly enriched microglial genes during development (n = 75). (C) Transcript reads per million expression levels of a selection of homeostatic and immune modulatory microglial markers during development. (D) Volcano plot of upregulated and downregulated microglial genes at the peak of differential gene expression between 9 and 10 pcw, with gene ontology analysis significant for cellular component. (E) Regional differential expression between time points of the cortical adult microglial genes. (F) Actively cycling and proliferating microglia in the scRNA-seq dataset were identified across the gestational age. Only ages with a minimum of 50 cells were selected (7–24 pcw). Cycling and proliferating cell signatures display a bimodal pattern of proliferation consistent with histological findings. (G) Metascape analysis of conserved cycling and proliferating microglia markers. (H) Gene ontology and protein-protein interaction enrichment analysis marking key mitotic cell-cycle processes. Protein-protein interaction enrichment identifies mitotic spindle checkpoint and amplification of signals from the kinetochores (in red), as well as mitotic chromosome condensation and condensing I complex (in blue). DE, differentially expressed genes.

We sought to validate our histological findings with an alternative approach based on the analysis of the transcriptional profile of microglia. Using bulk RNA-seq data from whole brain (Gerrelli et al., 2015; Lindsay et al., 2016), we examined the expression of genes characteristic of published adult and juvenile microglial signatures (Figure 5A, top panel; Data S1) across development (7–17 pcw). We considered a gene to be constitutively expressed when present in >80% of the samples in every time point at a TPM value > 2 (Figure 5A). We identified that 24% (212) of cortical genes from the adult signature were constitutively expressed in the telencephalon between 8 and 17 pcw ("On" genes; Figure 5A, bottom panel). These genes are involved in the regulation of the innate immune and inflammatory responses as well as cytokine production (Figure 5A, right panel). Heatmap representation of a set (75) of highly enriched microglial genes (Data S1) indicated a highly changeable signature with higher expression in the earlier time points (8 and 9 pcw) compared with later time points, where only a few genes had high expression by 13–17 pcw (Figure 5B). The homeostatic marker P2RY12 had a stable expression throughout, while CX3CR1 increased gradually toward the 13- to 17-pcw temporal window (Figure 5C). Pro-inflammatory factors, such as IRF5 and IFNGR1, had a stable expression throughout. The sensome gene CD37 had a 100-fold higher expression compared with other genes in our samples, declining with age. AIF1, encoding the ubiquitously expressed IBA1 protein, was expressed at low levels but consistently across the various ages (Figure 5C).

We identified a peak of differentially expressed (DE) genes at 9 pcw in the cortex (Figure 5D), matching the timing of the start of the first wave of expansion of microglial density (Figure 3). At 9pcw, we detected 81 DE genes: 27 upregulated DE genes were associated with chemokine and Toll-like receptor signaling, such as IRF5, SIGLECs (e.g., SIGLEC10), and MHC-II/co-stimulatory molecules such as HCG4B (Figure 5D; Data S1), and 54 downregulated DE genes were associated with cytokine-cytokine receptor signaling and interferon-gamma-mediated signaling such as TGFBR1 (Figure 5D). Collectively, microglial DE genes are significantly associated with cellular components of immunological synapses and cytoplasmic, as well as intracellular, vesicles (Figure 5D).

We compared the expression of the microglial signature by region, testing samples from the cerebellum, the choroid plexus, the cortex, and the midbrain (Figure 5E). We detected a substantial increase in the %DE genes in the midbrain and cerebellum at 7–8 pcw, prior to that seen in the cortex and choroid plexus between 9 and 10 pcw (Figure 5E). Although we did not perform deconvolution of cell-specific gene expression, this would suggest that different regions may follow different microglial maturation timings. Thereafter, change in gene expression by region declined substantially with age (Figure 5E).

To further our analysis, we performed an integration of 4 available datasets from scRNA-seq of human brain myeloid cells (Bian et al., 2020; Kracht et al., 2020; Fan et al., 2020; Cao et al., 2020). We also considered including single-cell data into our integrated single-cell dataset from (Li et al., 2018), as this study shows that deconvolution of tissue-level data against cell-type-enriched markers for microglia follows an inverse sigmoidal curve. However, given the number of single-cell microglial transcriptomes available (∼5%–10% of 1,195 cells are likely to be microglial), it was unlikely that the pattern of our data would have changed significantly if we had incorporated these data. As each of the individual datasets (Bian et al., 2020; Kracht et al., 2020; Fan et al., 2020; Cao et al., 2020) covered specific temporal windows, the integration allowed the analysis of an extended timeline (Table S1), similar to that analyzed by immunohistochemistry (IHC) (Figure 3) and bulk RNA-seq (Figures 5A–5E). The initial integration identified the presence of cells expressing markers of erythrocytes in the original datasets (Figure S5A), and we removed these and re-clustered to avoid any effects of contamination with non-myeloid cells (Figure S5C). In the integrated dataset, we identified 8 clusters of microglia that express typical myeloid markers (see cluster markers in Data S2), among which are actively cycling and proliferating cells (cluster 5) (Figures 5F and S5). The integration successfully represented all datasets, regardless of the original number of cells (Figure S5D). The proliferation cluster also displayed a wave-like distribution across ages, first peaking at 9 pcw and then peaking again at 18 pcw (Figures 5F and S5), tracking the pattern we observed at the histological level. The alignment of cycling and proliferative microglial cells between the source data identified 40 conserved markers (Data S2). Gene ontology and protein-protein interaction enrichment analyses of these genes underscored their association with cell-cycle processes (Figures 5G and 5H), supporting the correct identification of these cells as proliferative microglia.

### Microglial proliferation is prominent in key neurodevelopmental structures

The fetal SP is a major site for synaptogenesis and neuronal maturation, as well as a waiting compartment for cortical afferents (Kostović, 2020). Microglia are enriched in the PSP monolayer below the CP from the 10^th^ pcw (Figures 3A and 4A). The SP is fully formed at 12.5 pcw by the gradual merging of the PSP and the deep, loose portion of the CP in parallel with the development of the afferent fiber system (Kostović, 2020). Microglia appear to track these key changes, appearing densely clustered in the SP, with microglial proliferation highest in the SP and the IZ (Kruskal-Wallis test, adjusted p < 0.01) (Figures 4E and S4A–S4F).

By 15–20 pcw, we observed significant clustering of microglia in the periventricular crossroads of projection and callosal pathways in the frontal lobe; these cells co-express MHCII and are non-proliferative (Figures S4C–S4E). By 20 pcw, microglia are evenly distributed throughout the expanding SP and only penetrate the upper portion of the CP by 25 pcw (Figure S4B). Dense clusters of microglia can be observed in the VZ from 22 to 28 pcw, colonizing all the cortical layers by 28 pcw (Figure S4B). From 32 pcw, the 6-layered cortical Grundtypus can be observed with a resolving SP zone, and gray matter (GM) and white matter (WM) are clearly identifiable.

In sum, we found qualitative evidence of the intimate association of microglia with key neurodevelopmental hallmarks that characterize brain formation and wiring.

### The microglial population undergoes an expansion phase during infancy and childhood and is refined to adult levels by cell death

**Figure 6:**
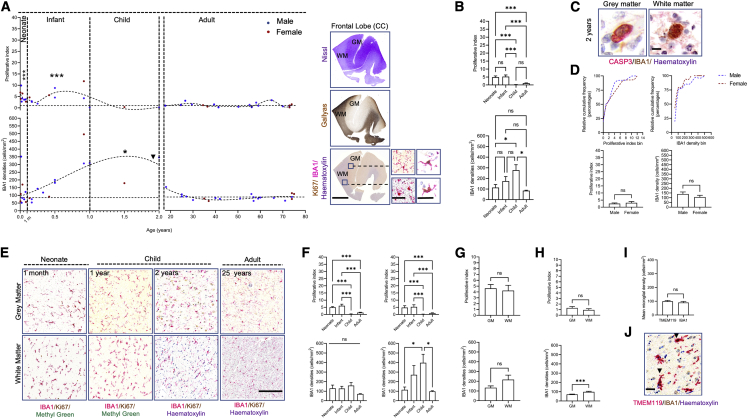
Postnatal dynamics of microglia in the cortex (A) Microglial densities and proliferative dynamics in early postnatal life (n = 24; 14M:10F) and adulthood (n = 21; 14M:7F) and a representative cross-section of frontal cortex with anatomical histochemistry and photomicrographs of homeostatic and proliferating microglial morphologies in gray and white matters. Neonatal and infant proliferation is most significant against other postnatal windows, while microglial densities are most significant in the child against other temporal windows. Scale bars: 2 mm (left); 40 μm (right). (B) Mean proliferation and mean densities in the neonate, the infant, child, and adult. Data are shown as mean ± SEM. (C) Microglial cell death in gray and white matters at 2 years (black arrow in A). Scale bars: 15 μm. (D) Cumulative relative frequency distribution plots of microglial densities and proliferation by sex (top panel) and mean proliferation and densities by sex (28M:17F) (bottom panel, data are shown as mean ± SEM). (E) Representative photomicrographs of cell densities in gray and white matters from birth to 2 years. Scale bars: 500 μm. (F) Regional differences of microglial densities and proliferation in gray (left) and white matters (right) between the neonate, infant, child, and adult (n = 45). Data are shown as mean ± SEM. (G) Proliferation rates and densities in early postnatal life in gray and white matters (n = 24). Data are shown as mean ± SEM. (H) Regional differences in the adult in gray and white matters in proliferation (top) and density (bottom). Data are shown as mean ± SEM. (I) Mean TMEM119 and IBA1 cell densities postnatally (n = 5) (left) and ratio of TMEM119/IBA1 between early life (3–6 months, n = 2) and adult life (40–70 years, n = 3). Data are represented as mean ± SEM. (J) Representative photomicrograph of TMEM119/IBA1 labeling showing colocalization of both markers (black arrows). Scale bars: 25 μm. CC, cingulate cortex; GM, gray matter; WM, white matter. For all panels, asterisks represent adjusted p value significance as follows: ^∗^p < 0.05, ^∗∗^p < 0.001, ^∗∗∗^p < 0.0001, and ns p > 0.05.

Postnatally, the microglial density increases from birth, and at around 1–2 years of age we observe a third wave. Though not statistically significant (bimodal distribution excess test p > 0.05), we thought it important to characterize, given the challenge of obtaining samples during the crucial temporal window of early postnatal life, particularly infancy and childhood. Furthermore, the pattern observed strikingly resembles the developmental pattern. This third wave is characterized by a significant increase in proliferation during neonatal and infant life compared with adulthood and childhood (Kruskal-Wallis p < 0.0001, adjusted p values < 0.001) (Figures 6A and 6B). This is followed by a 3- to 4-fold increase in densities in childhood in comparison with neonatal and adult densities (Kruskal-Wallis p < 0.01, adjusted p values < 0.01) (Figures 6A and 6B). In childhood, proliferation decreases to levels observed in the adult, remaining constant through life (Figures 6A and 6B) (p > 0.05 between adulthood and childhood). The mechanism through which microglial densities decrease after 1.5 years may be likened to the selection phase observed in rodents postnatally (Askew et al., 2017; Nikodemova et al., 2015), and we indeed detect microglial death (IBA1^+^caspase-3^+^) in the human cortex (Figure 6C). We found no significant differences between the cumulative distributions by sex for both density and proliferation (Kolmogorov-Smirnov test, p > 0.05) (Figure 6D) or when comparing mean proliferation and densities by sex during postnatal life (0–75 years) (Mann-Whitney U test, p = 0.09 for densities, p > 0.2 for proliferation). Regionally, mean GM and WM proliferative indices are similar in early postnatal life (neonate–child) (Figures 6E–6G) and WM densities are higher but not significant statistically (Figure 6F). WM densities, however, are highest in the child compared with the infant, neonate, and adult (Figures 6E and 6F) (Kruskal-Wallis p < 0.01, adjusted p < 0.05). The adult density and proliferation of microglia retain the previously described (Mittelbronn et al., 2001) higher density in the WM (Figures 6G and 6H). From 18 years of age, throughout adult life and healthy aging, the population has a slow turnover of 1.12% ± 0.18% and the density stabilizes at 84.63 ± 4.42 cells/mm^2^ (Figure 6H). The mean TMEM119 and IBA1 densities are similar during postnatal life (Figures 6I and 6J) (Wilcoxon test, p > 0.05). Our TMEM119 findings were consistent with mouse studies showing limited/absent expression during the developmental period and an increased expression in postnatal life (Bennett et al., 2016). These analyses collectively identify that infancy and childhood are important temporal windows for microglial dynamics.

## Discussion

The past decade saw an exponential increase in experimental studies focusing on microglia, in part fueled by the resolution of a long debate on the origins of these cells in mouse by fate-mapping studies (Ginhoux et al., 2010). In humans, microglia enter the brain primordium prior to the onset of substantial neurogenesis and neuronal migration (Menassa and Gomez-Nicola, 2018). Recent studies indicate that microglia show marked heterogeneity during development, becoming immunocompetent from the 10^th^ pcw (Kracht et al., 2020), with conserved ontogenic pathways between mouse and human (Bian et al., 2020). The population expands during embryonic life to yield the adult population, which maintains itself by cycles of self-renewal at a slow daily turnover rate (Askew et al., 2017; Réu et al., 2017; Nikodemova et al., 2015). Due to the limited availability of healthy human developmental tissue, most of our knowledge of microglial development stems from rodent studies. Such studies have highlighted the roles that microglia play in shaping the neurodevelopmental landscape, including neuronal genesis, migration, dendritic development, axonal pruning, synaptogenesis, and wiring (Paolicelli et al., 2011; Squarzoni et al., 2014; Verney et al., 2010). However, until now we lacked a reference map for the development of microglia in humans as an essential framework to contextualize basic studies and target human-relevant mechanisms.

Here, we have characterized the precise spatiotemporal dynamics of microglia in the frontal cortex across the human lifespan (3^rd^ pcw—75 years). We provide granularity and breadth, identifying critical temporal windows during development and postnatal life and defining processes fundamentally different from the known development of murine microglia, which will influence how we study the role of these cells in humans. This was achieved by the collection of post-mortem specimens from a multitude of tissue resources, enabling us to conduct a cross-sectional study with an extensive scope. We determine that microglia enter the telencephalon by the 4^th^ pcw, mirroring, with some delay, the colonization of peripheral organs by other tissue-resident macrophages. After colonization, microglia undergo significant fluctuations in their cell density, characterized by newly identified wave-like patterns of proliferation followed by apoptosis. This is particularly evident at a key neurodevelopmental stage, the transition between embryonic and fetal life (9^th^ pcw). This pattern is corroborated by bulk RNA-seq and scRNA-seq analyses and coincides with the appearance of the CP and transient zones. Throughout embryonic, fetal, and postnatal development, the population transits through different cycles of expansion and apoptosis-driven refinement, stabilizing during childhood and being maintained by self-renewal across the adult and older ages.

The elucidation of microglial dynamics during human cortical development offers new avenues for examining how these cells contribute to neurodevelopmental disorders. These cells participate in brain wiring pre- and postnatally (Menassa and Gomez-Nicola, 2018; Squarzoni et al., 2014; Paolicelli et al., 2011) and are involved in the pathophysiology of autism spectrum disorder (Tetreault et al., 2012; Carroll et al., 2021), schizophrenia (Sekar et al., 2016), and intellectual disability (Coutinho et al., 2017). With our identification of critical temporal windows of the population’s expansion and refinement, we pave the way for uncharted territories in the field of neurodevelopmental disorders, which, with the current tissue resources in place, may begin to closely characterize in space and time how the population alters the neurodevelopmental environment. We know that targeting microglial dynamics may have a therapeutic potential when these cells go awry, and this is likely to extend to the treatment of neurodevelopmental disorders (Olmos-Alonso et al., 2016).

One interesting, and somewhat unexpected, finding of this study was the very limited, and in most cases absent, sex-dependent differences in the different variables we analyzed related to microglial development. This is in contrast to previous rodent literature, which observed increased microglial density in males postnatally, as well as sex-dependent morphological changes lasting until juvenile and adult stages (Schwarz et al., 2012). Differences between developing microglia in male and female rodents have also been framed within the microglial developmental index (MDI), a compound index of transcriptional maturation. This program was found to be delayed in males relative to females and sensitive to immune challenge (Hanamsagar et al., 2017). This perhaps underscores the urgent need to pivot to human or humanized models in order to understand the biology of microglia and thus refine our knowledge of the contribution of this myeloid population to human brain function and dysfunction.

Altogether, our findings identify key features of, and relevant temporal windows for, microglial population expansion and refinement across the human lifespan. This study provides a foundational map of the precise microglial spatiotemporal dynamics from early development through adulthood to healthy aging. This resource will inform future research on how microglial cells participate in the neurodevelopmental landscape in humans and their relevance for neurodevelopmental disorders, as they form part of the pathological signature of these conditions (Velmeshev et al., 2019; Coutinho et al., 2017).

### Limitations of the study

Our study was affected by the scarcity of post-mortem brain samples from key ages. For example, we could not obtain tissues from childhood and adolescence, a period of protracted intracortical myelination in the frontal and temporal cortices in humans (Deoni et al., 2015). As we know, microglia follow myelination very closely (Menassa and Gomez-Nicola, 2018; Verney et al., 2010); the dynamics of the population have yet to be carefully defined during the critical window of intracortical myelination in humans. Early adolescence is a dynamic period and is important for the onset of some neuropsychiatric disorders (Penzes et al., 2011). Additionally, hormonal changes during puberty are likely to influence microglial dynamics and these could drive sex-specific effects. Another limitation relates to our definition of migration, which is inferred from morphometric analyses that may be less accurate in reflecting true microglial movement and did not allow us to distinguish between peripheral migration (into the brain) and within-brain migration. Microglia acquire a migratory phenotype subsequent to a proliferation cycle (Martín-Estebané et al., 2017), and our assessments here are of a possible migratory phenotype irrespective of contribution. We observed that within-brain migration may vary according to the topography of transient layers, defined by the type of neurogenetic process. Overall, migration may contribute to the growth of the population at specific time points during development; thereafter, and once microglia are in the parenchyma and proliferating, they can acquire a migratory phenotype after each proliferative cycle to tile the anatomical local territory for which they are destined.

## STAR★Methods

### Key resources table

**Table:** 

REAGENT OR RESOURCE	SOURCE	IDENTIFIER
**Antibodies**		

Rabbit anti-IBA1	Wako Chemicals	Cat# 019-19741
Goat anti-IBA1	Abcam	Cat# ab5076
Mouse anti-IBA1	Abcam	Cat# ab283319
Mouse anti-MHC-II/HLA-DP/DQ/DR	Dako, Agilent	Cat# M077501-2
Rabbit anti-PU.1	Cell Signalling	Cat# 2258S
Biotinylated anti-RCA1	Vector Labs	Cat# B10855
Rabbit anti-TMEM119	Abcam	Cat# ab185333
Mouse anti-CD68	Dako, Agilent	Cat# GA609
Mouse anti-SOX2	Santa Cruz	Cat# sc365823
Mouse anti-Ki67	Dako, Agilent	Cat# M724029-2
Rabbit anti-Caspase3 (cleaved)	Cell Signalling	Cat# 9664S
Goat anti-rabbit Alexafluor 488	Invitrogen	Cat# A32731
Goat anti-mouse Alexafluor 568	Invitrogen	Cat# A11031
Horse anti-rabbit HRP (DAB, brown)	Vector Labs	Cat# SK4103
Horse anti-mouse HRP (DAB, brown)	Vector Labs	Cat# MP7724
Horse anti-rabbit HRP (DAB + Nickel, black)	Vector Labs	Cat# SK100
Horse anti-rabbit AP (magenta)	Vector Labs	Cat# MP7724
Horse anti-rabbit AP (blue)	Vector Labs	Cat# SK5400

**Biological samples**		

Human post-mortem developmental tissues	Wellcome/MRC HDBR	N/A
Human post-mortem developmental tissues	Zagreb Brain Collection	N/A
Human post-mortem developmental tissues	Thomas Willis Oxford Brain Bank	N/A
Human post-mortem developmental tissues	Southampton Brain Bank	N/A
Human post-mortem postnatal tissues	Southampton Brain Bank	N/A
Human post-mortem postnatal tissues	Edinburgh Brain Bank	N/A
Human post-mortem postnatal tissues	Cambridge Brain Bank	N/A
Human post-mortem postnatal tissues	Southwest Dementia Brain Bank	N/A

**Chemicals, peptides, and recombinant proteins**		

Immpress Duet Kit	Vector Labs	Cat# MP7724
ImmPACT DAB-EqV substrate kit	Vector Labs	Cat# SK4103
BCIP/NBT substrate kit	Vector Labs	Cat# SK5400
Vectastain Elite ABC kit	Vector Labs	Cat# PK6100
Haematoxylin QS	Vector Labs	Cat# H3404-100
Methyl Green	Vector Labs	Cat# H3042
Dual enzyme block	Dako, Agilent	Cat# S2003
Periodic Acid	Sigma-Aldrich (Merck)	Cat# P7875
Schiff’s Reagent	Sigma-Aldrich (Merck)	Cat# 3952016
Alcian blue	Sigma-Aldrich (Merck)	Cat# A5268
Cresyl Violet acetate	Sigma-Aldrich (Merck)	Cat# C5042
Citric acid	Sigma-Aldrich (Merck)	Cat# 251275
Sodium borohydride	Sigma-Aldrich (Merck)	Cat# 213462
Vectashield antifade mounting medium	Vector Labs	Cat #H1000-10
DPX mounting medium	Sigma-Aldrich (Merck)	Cat # 06522
Trueblack autofluorescence quencher	Biotium	Cat# 23007

**Deposited data**		

Bulk human RNAseq data	This paper	Gerrelli et al., 2015 & Lindsay et al., 2016
Human adult microglial juvenile RNAseq data	This paper	Galatro et al., 2017
Human adult microglia adult RNAseq data	This paper	Gosselin et al., 2017
Human development single-cell RNA seq	This paper	Bian et al., 2020
Human development single-cell RNA seq	This paper	Kracht et al., 2020
Human development single-cell RNA seq	This paper	Fan et al., 2020
Human development single-cell RNA seq	This paper	Cao et al., 2020

**Software and algorithms**		

Prism 9	Graphpad	https://www.graphpad.com/ scientific-software/prism/
Fiji	Schindelin et al., 2012	https://imagej.net/software/fiji/
R studio	R Studio Development Team	https://www.rstudio.com/
QGIS (v2.18.3)	QGIS Development Team	https://www.qgis.org/en/site/
Seurat (v3.2.2)	Stuart et al., 2019	https://satijalab.org/seurat/
Fluoview Olympus	Olympus	https://www.olympus-lifescience.com/en/laser-scanning/fv3000/
Imagescope Aperio (v12.4.3)	Leica biosystems	https://www.leicabiosystems.com/en-gb/digital-pathology/manage/aperio-imagescope/
VS110 Desktop (v4)	Olympus	https://www.olympus-lifescience.com/en/solutions-based-systems/vs200/
NDP.view2	Hamamatsu Photonics	https://www.hamamatsu.com/jp/en/product/life-science-and-medical-systems/digital-slide-scanner.html
Gimp (v2.10.22)	Gimp Development Team	https://docs.gimp.org/2.10/en/
Samtools (v1.1)	Li et al., 2009	http://samtools.sourceforge.net/
EdgeR	Robinson et al., 2010; Soneson and Robinson, 2018	https://bioconductor.org/packages/release/bioc/html/edgeR.html
HTSeq (v0.6.1)	Anders et al., 2015	https://academic.oup.com/bioinformatics/article/31/2/166/2366196
Trinity (v2.4.0)	Haas et al., 2013	https://www.nature.com/articles/nprot.2013.084
Metascape	Metascape Development Team	https://metascape.org/gp/index.html#/main/step1
STAR v2.5.2b	Dobin et al., 2013	https://academic.oup.com/bioinformatics/article/29/1/15/272537?login=false

### Resource availability

#### Lead contact

Further information and requests for resources and reagents should be directed to and will be fulfilled by the Lead Contact, Diego Gomez-Nicola (d.gomez-nicola@soton.ac.uk).

#### Materials availability

This study did not generate new unique reagents.

#### Data and code availability

• The integrated human single-cell RNA-seq dataset, and the developmental layer isolation macro are publicly available in [Synapse]: [syn33055573].
• Code is available in [Synapse]: [syn33055573].
• Raw data is available upon request to the Lead Contact.

### Experimental model and subject details

#### Human tissues

Human developmental tissues were obtained through the joint Medical Research Council (MRC)/Wellcome Trust Human Developmental Biology Resource (HDBR) (Gerrelli et al., 2015), the Zagreb Research Brain Collection (Hrabač et al., 2018) and BRAINUK neuropathology centres (Figure 1; Table S2 for demographics of cases and acknowledgements for participating BRAINUK centres). Tissues were collected with appropriate maternal consent and approval for use in research. Ethical approval was obtained from the relevant ethics committees (Newcastle and North Tyneside NHS Health Authority Research Ethics Committee, Fulham NHS Health Authority Research Ethics Committee, South-Central Oxford C NHS Health Authority Research Ethics Committee and the South-Central Hampshire B NHS Health Authority Ethics Committee). Embryonic ages were estimated according to the Carnegie classification (CS) provided by the HDBR, CS23 being the last Carnegie stage equivalent to the 9^th^ pcw. Gestational age corresponds to the time elapsed between the first day of the last menstrual period and the day of delivery (Engle et al., 2004). We used here postconceptional age consistent with HDBR guidelines which is estimated at 2 weeks earlier than gestational age (Table S2). For fetal developmental stages (age>9 pcw until term), cases were aged by a neuropathologist according to clinical notes. When possible, developmental cases were sex-matched by timepoint. 63 developmental cases were initially sampled but only 52 developmental cases were included in the final study (n = 15 embryonic tissues; n = 37 fetal tissues; Figure 1 and Table S2) between the late 3^rd^ pcw (CS10) and 38 pcw (term). We excluded cases due to poor immunoreactivity (poor antigenicity) or evidence of hypoxic injury identified in the clinical notes (Figure S6A; Table S2). Where possible, maternal data were provided (Table S3). Exclusion criteria, assessed against individual medical histories, included brain injury due to hypoxia-ischaemia or trauma, infection, or genetic mutations affecting brain structures. 71 postnatal cases were collected between 0 and 75 years of age with 45 of these cases in the final study. Early postnatal brain tissues (n = 24) aged between 0-2 years were obtained through BRAINUK. Adult brain tissues (n = 21) aged between 18-75 years were obtained with informed written consent and material approved for use for research purposes through the Zagreb Brain Collection, Edinburgh Brain Bank, the Cambridge Brain Bank and the Southwest Dementia Brain Bank. Ethical approvals were from the East Scotland NHS Research Ethics Service, the Cambridgeshire NHS Health Authority Research Ethics Committee and the North Somerset and South Bristol NHS Health Authority Research Ethics Committee, respectively. It was not possible to get sex-matching in postnatal cases (n = 45) and additional exclusion criteria included any neurological co-morbidity or a diagnosis of a connectivity disorder (e.g., autism spectrum disorder) (Table S4). We could not obtain samples from children aged between 3 and 17 years as these were scarce and, when present, the absence of consent prohibited the usage of these tissues for research.

All tissues were received as paraffin-embedded slices previously fixed in formalin phosphate buffer saline and sometimes with an added secondary fixative, methacarn (HDBR samples). Mid-sagittal embryonic sections of 8 μm thickness were obtained through the whole embryo (up to CS21) allowing the visualisation of the brain rudiment and organs such as the heart, liver and spleen. All other sections, from CS23 until the late postnatal ages were processed coronally through the frontal axis of the brain at a thickness of 8 μm.

#### Anatomy and neuropathology

Embryonic samples along the midsagittal axis included the telencephalic wall with its dorsal and ventral components. The frontal cortex (dorsolateral prefrontal, anterior cingulate) develops from the dorsal telencephalon (Bayer and Altman, 2008; Bayer and Altman, 2006). After CS21, the medial GE in the ventral telencephalon becomes more prominent. From CS23 onwards, developmental sections were all along the coronal axis of the frontal lobe with a prominent medial GE, an expanding telencephalic wall featuring transitional zones: the MZ, CP, PSP, SP, IZ, SVZ, VZ; Figure S1C). By 32 pcw, frontal cortex grey and the underlying white matters are developed, transitional zones have largely resolved, and brain structures begin to resemble those from adulthood. All sections were histochemically labelled with haematoxylin and eosin according to standard methods, assessed by a neuropathologist for any signs of tissue pathology prior to analysis such as local or generalised hypoxia, haemorrhage, gliosis or neuronal death. Developmental tissue sections were histochemically labelled with filtered cresyl violet for the Nissl substance to visualise transitional zones for anatomical delineation as specified elsewhere (Duque et al., 2016; Kostović et al., 2002). The SP was visualised by histochemical labelling of the extracellular matrix using the Periodic Acid Schiff Reagent-Alcian Blue method as specified elsewhere (Kostović, 2020; Kostović et al., 2002). White matter was visualised using the Gallyas silver labelling method by means of physical development (Gallyas, 1979).

### Method details

#### Immunohistochemistry

For immunostaining, sections were placed in an oven at 60°C for 45 min, dewaxed in 100% xylene solution, rehydrated in decreasing graded absolute ethanol (in distilled water) solutions and washed in water and 0.1% TWEEN 20-phosphate buffer saline (PBS-T) solution. Heat-mediated antigen retrieval in 10 mM citrate buffer (pH=6.2) was subsequently performed on the sections in a microwave for 25 min. For early timepoints (CS10-CS13), 30 min antigen retrieval was performed using 0.5% sodium borohydride (213462, Sigma-Aldrich, UK). Then, sections were washed and/or cooled in cold tap water. Endogenous peroxidase and phosphatase activity in the tissues was quenched with a dual enzyme block from Dako (S2003, Agilent, UK). Sections were washed in 0.1% PBS-T and blocked with the relevant serum (goat, horse or rabbit) and 5% bovine serum albumin (BSA) in 0.2% PBS-T for one hour. Microglia were probed with a 24h incubation at 4ºC with either a rabbit anti-human IBA1 antibody diluted in blocking solution (1:200, 019-19741, Wako chemicals, USA) or a goat anti-human IBA1 antibody (1:200, ab5076, Abcam, UK) or a mouse anti-human IBA1 antibody (1:200, ab283319, Abcam, UK). We used additional microglial markers including mouse anti-MHC-II/HLA-DP/DQ/DR (1:250, M077501-2, Agilent, UK), rabbit anti-PU.1 (1:200, 2258S, Cell Signalling, UK), biotinylated RCA1 (1:1000, B10855, Vector Labs, UK), rabbit TMEM119 (1:500, ab185333, Abcam, UK). We also used mouse an anti-human CD68 as a lysosomal marker (1:400, GA609, Dako, Agilent, UK) and a mouse anti-human SOX2 antibody (1:200, sc365823, Santa Cruz Biotechnology, USA) as a neuronal precursor cell marker. Mouse anti-Ki67 (1:400, M724029-2, Agilent, UK) was used to label proliferative cells and rabbit anti-cleaved-Caspase3 (1:40, 9664S, Cell Signalling, UK) was used to label apoptotic cells. Following incubation with the primary antibody, sections were washed with 0.1% PBS-T and incubated with the appropriate secondary antibodies. Secondary antibody double labelling was through an ImmPress Duet double staining anti-mouse HRP (brown)/anti-rabbit AP (magenta) kit (MP7724, Vector Laboratories, UK) according to the manufacturer’s instructions; horse anti-rabbit HRP using DAB chromogen with Nickel (SK100, Vector Laboratories, UK); horse anti-rabbit AP (blue) using a BCIP/NBT substrate kit (SK5400, Vector Laboratories, UK); rabbit anti-mouse secondary using the ImmPACT DAB-EqV HRP (brown) substrate kit (SK4103, Vector Laboratories, UK); signal amplification after species-specific secondary biotinylated antibody incubation of two hours was done using an avidin/biotin-based peroxidase system (PK6100, ABC Vectastain Elite kit, Vector Laboratories, UK). Chromogen development was sequential with double labelling. For triple labelling in brightfield, an additional DAB chromogen development was used, sections were re-quenched, re-blocked and probed with the relevant primary and secondary antibodies (bound to HRP) then developed with DAB + Nickel (SK4100, Vector Laboratories, UK). Chromogen development reactions were halted with distilled water, sections were washed with 0.1%PBS-T for 15 min then counterstained with methyl green (H3042, Vector Laboratories, UK) for 10 min or haematoxylin QS (H3404-100, Vector Laboratories, UK) for 30 seconds diluted 1:3 in distilled water. Sections were subsequently washed in distilled water for 5 min, then dehydrated in increasing gradients of absolute ethanol, cleared in xylene for 15 min, coverslipped with permanent mounting medium (DPX), dried for 24 hours then cleaned for scanning.

#### Immunofluorescence

For immunofluorescence, we followed the same protocol as our brightfield immunohistochemistry except for the following steps: no blocking needed against endogenous peroxidases and phosphatases. Secondary incubation against primary targets was done with the relevant secondary antibodies conjuguated to fluorophores (Goat-anti rabbit Alexafluor 488, 1:500 (Invitrogen, UK) and goat anti-mouse Alexafluor 568, 1:500 (Invitrogen, UK)) for 2 hours at room temperature. Slides were subsequently washed with PBS-T(0.1%) and incubated to quench autofluorescence with Trueblack lipofuscin autofluorescence quencher (23007, Biotium, USA) at 1:100 in 70% ethanol for 1 minute. Slides were washed with PBS-T(0.1%) and incubated for 5 min in 1:50000 DAPI in PBS then washed and coverslipped in Vectashield antifade mounting medium (H1000-10, Vector Laboratories, UK) in preparation for confocal imaging.

#### Confocal imaging

Tissues labelled with fluorophores were scanned using an Olympus FV3000 confocal microscope (Olympus, Europe) with a 20x objective (UPlanSApo, NA 0.75, Olympus). Z-stacks were taken every 5 um for a total of 10-20 optical sections then Z-projected in imaFV31S-SW Fluoview software to reveal morphology. Pixel resolution was 0.45 um x 0.45 um in (x, y). All images were imaged in three channels using the 488, 568 and 350 nm laser lines.

#### Image analysis

Slides were digitised using a VS110 high-throughput virtual microscopy system (Olympus, Japan), an Aperio Scanscope AT Turbo system (Leica Biosystems, UK) or a 2.0 RS Nanozoomer high-throughput system (Hamamatsu Photonics, Japan). Pixel resolution was 172.35 nm/pixel in x, y and z. From the scans, regions of interest were extracted, and brain/layer thicknesses were measured in the relevant slide viewers (VS110-Desktop (v4), Aperio ImageScope (v12.4.3), NDP.view2). Images were processed using Fiji (Schindelin et al., 2012). The analysis pipeline included re-scaling the images, deconvolving the signal and calculating a cell density (in cells/mm^2^) based on absolute counts until CS23. Beyond that age, between 500-1000 cells/case were sampled. While stereological methods could not be used due to the limited tissue available as well as the thickness of sections obtained, this approach was deemed sufficient to provide a representative value of brain IBA1 densities consistent with unbiased sampling methods (Herculano-Houzel et al., 2015). The proliferative index -I- of microglia was calculated as follows: I = (number of double positive (IBA1/Ki67) cells/total number of IBA1 cells) x 100 (see also (Askew et al., 2017)). Double positives were confirmed by deconvolution (Figure S6B). Prenatally, we calculated fold change values between subsequent ages based on overall cortical wall or individual layer thickness measurements to account for brain/layer growth rates during development. Postnatally, we collected brain weight data (when available) from the clinical notes of analysed cases and calculated the fold change increase in brain weight between subsequent ages and applied this correction to our density data. Proliferation index was independent of the changes in brain growth rates. Individual layers were manually traced in Fiji using a macro (Synapse ID: syn33055573) and a cell density was calculated as N_D_ = (Number of cells/area (mm^2^)).

#### Analysis of TMEM119/IBA1 cells

In a subset of cases (n = 15, 10 prenatal and 5 postnatal) and along the frontal neocortical wall, we calculated the density of TMEM119^+^ and IBA1^+^ cells and the ratio between TMEM119^+^/IBA1^+^ densities to determine the percentage labelled by each marker. We also performed colocalisation analyses using two methods in brightfield: the colocalisation algorithm in Aperio imagescope (Leica Biosystems, UK) and using the deconvolution plugin in Fiji software (Figure S3B).

#### Calculation of the apoptotic index

We quantified IBA1^+^ cells positive for active cleaved Caspase 3 as a marker of cell death. Apoptotic cells undergo nuclear and cytoplasmic degradation that can be visible under haematoxylin and eosin histochemistry too. Cells show eosinophilic changes in the cytoplasm early in the process with a pyknotic nucleus. Further on, the nuclear envelope disintegrates, and the eosinophilic cytoplasm shrinks before the cell undergoes karyorrhexis or the breakdown of nuclear material altogether (Sierra et al., 2013; Love et al., 2015). We calculated the apoptotic index for a selection of cases around the main peak of the most significant increase in microglial densities (n=15; 8 pre-peak between 7-11 pcw and 7 post-peak between 12-16 pcw). The index was calculated as a percentage of double positive cells for cleaved caspase 3 and IBA1 against the total number of IBA1^+^ cells. Overall, if we approximated the total number of cells lost per day per square mm based on our mean density measurements after the peak, we could only account for a fraction of the lost cells per week (approximately 9 cells per square mm). Given the rapid tagging of a cell with active caspase 3 (∼5 min) ((Sierra et al., 2013), which may be further compounded by sensitive antigenicity in formalin-fixed paraffin-embedded tissues, it is possible that the index is an underestimation.

#### Migration analysis

The migratory phenotype of microglia (IBA1^+^ cells) was inferred using morphometric analysis in Fiji (Schindelin et al., 2012). While dynamic processes such as migration cannot be easily assessed in static images, we used morphological determinants to determine the possible migratory status of microglia based on studies that have linked microglial phenotype to their migratory state in different stages of development (Harry, 2013; Sánchez-López et al., 2004; Martín-Estebané et al., 2017; Marín-Teva et al., 1999; Marín-Teva et al., 1998; Cuadros and Navascués, 1998; Rezaie and Male, 1999; Swinnen et al., 2013). Microglia in adult brain are not polarised since they are already differentiated into their final and ramified morphology (thin and long branches). However, during development, microglia are amoeboid and they show short but thick pseudopodia which they use to migrate along fibres of radial glia, white matter systems and blood vessels. These two morphologies are very different, and they can be found in different developmental stages. In quail, these have been described alongside the movement of microglia using pseudopodia, which is a similar phenotype to the one we observed in the human samples: cells with thick processes that are very polarised in parallel to fibre layers or radial glia along the layers. The circularity parameter was used to select out the migratory phenotype in each layer: if circularity<0.3, a cell would be considered migratory and if circularity>0.3, it would be non-migratory (Figure S6C). We also assessed the type of migration (radial or tangential) by measuring the angle formed between the major axis of the cell and the corresponding layer plane (Figure S6C), considering an angle<45 degrees as tangential migration and an angle>45 degrees as radial migration. Developmental timepoints considered were selected based on the cell density profile whereby an increase could not be explained by proliferation alone. We studied migration between 5 and 26 pcw (n=2/timepoint).

#### Histological heatmaps

For the visualization of cell densities as heatmaps, cortical columns per stage were manually annotated in Aperio ImageScope software (v12.4.3), LeicaBiosystems, IL, USA) (Figure S6D). At least 2 cases by timepoint were analysed and the most representative column was used for visualisation in the final spatiotemporal profile. IBA1^+^ and IBA1^+^/Ki67^+^ double-positive cells were analysed separately. Coordinates of the annotated cells were exported into Quantum Geographic Information System QGIS (v2.18.3, Hannover, Germany), a spatial analysis software. The heatmap module in QGIS with metric projection EOV23700 was used. We used a sampling radius of 150 μm, with a maximum value of 4, which meant that on the red area of a computed heatmap in a (0,15)^2∗^*π* mm^2^ area of a circle, at least 4 cells could be located. Subsequently, proportionally extrapolated values were used to estimate the density in cells/mm^2^. Heatmaps were exported and superimposed onto their respective immunohistochemically labelled spatial maps using Gimp 2.10.22 annotating the anatomical boundaries.

#### Bulk RNA-sequencing analysis

We sourced published lists of genes from adult and adolescent human cortical microglia (Galatro et al., 2017; Gosselin et al., 2017). 906 microglia genes were identified (Data S1) and were mapped onto an available bulk-RNAseq dataset from the HDBR. We utilised 251 samples of tissues ranging in age from 7 to 17 pcw. Four anatomical regions were considered: telencephalon (n = 94), cerebellum (n = 79), choroid plexus (n = 28) and midbrain (n = 50). Full details of the source, collection, preparation and sequencing of human fetal RNA samples have been described previously (Gerrelli et al., 2015; Lindsay et al., 2016) (https://www.hdbr.org/expression/). Data were downloaded from SRA as FASTQ files. Quality control was carried out using Trimmomatic to remove poor quality bases, reads with too many poor-quality bases and short reads using the following settings:*ILLUMINACLIP:/local/software/trimmomatic/0.32/adapters/TruSeq3-PE-2.fa:2:30:10 LEADING:5 TRAILING:5 SLIDINGWINDOW:4:15 MINLEN:72*. After this, unpaired reads were excluded from further analysis. STAR v2.5.2b (Dobin et al., 2013) was used to map reads back to the GRCh38 genome using settings --outFilterMismatchNmax 10 --outFilterMismatchNoverReadLmax 0.05 and the result outputted as .sam files, which were sorted using Samtools (v1.1, (Li et al., 2009)). Read counts were calculated per gene using HTSeq count (in the HTSeq v0.6.1 package; (Anders et al., 2015)) and the GRCh38 general feature format file. Differential gene expression analysis was carried out using EdgeR (Robinson et al., 2010; Soneson and Robinson, 2018) in Trinity (v2.4.0) (Haas et al., 2013) on raw read counts. Heatmaps were generated using *analyze_diff_expr.pl* in Trinity (Haas et al., 2013) and the TMM-normalised counts. Gene Ontology (GO) terms over-represented in the list of DE genes were identified using the plug-in available at the Gene Ontology resource.

#### Single-cell RNA-sequencing analysis

To validate our histological and bulk-RNAseq findings, we analysed 4 datasets from recently published developmental single-cell RNA-sequencing studies of human brain cells (Cao et al., 2020; Fan et al., 2020; Bian et al., 2020; Kracht et al., 2020) and generated an integrated dataset spanning 3 to 24 pcw (Bian et al., 2020; Kracht et al., 2020). We accessed the gene-cell count matrix and cell annotation matrix data and used Seurat (v3.2.2) for all analyses (Stuart et al., 2019). Guided by the original authors’ annotations, we enriched for microglial cells by selecting for “Immune”, “Mac_1”, “Mac_2”, “Mac_3”, “Mac_4”, and “Microglia”-annotated clusters. We then utilized the 3 x Mean Absolute Deviation (MAD) for outlier cut-off across 4 parameters where available: nCount_RNA, nFeature_RNA, percent.mt, percent.rb (Kracht et al., 2020; Daniszewski et al., 2018; Waise et al., 2019). After quality control, the final integrated dataset used for our analyses contained 24,751 transcriptomes, 8,117 of which were nuclei. Standard Seurat SCTransform integration was performed, selecting 3,000 features for anchor identification and integration and regressing for nCount_RNA, nFeature_RNA, percent.mt, and/or percent.rb (Figure S5). We detected immediate-early gene expression suggestive of dissociation-induced artefacts. However, a discussion of these effects in human tissue was beyond the scope of the study and were not selected for regression. We also identified a cluster enriched for erythrocyte (ERY) markers (*e.g.* HBG2, HBB) (Figure S5). The single-nuclei dataset by (Cao et al., 2020) contributed most of the cells of this ERY cluster, suggesting that this cluster is a technical, method-specific artefact, albeit all datasets showed some degree of ERY (Figure S5). We considered regressing for such effects, however, doing so risks distorting transcriptional heterogeneity. To minimize these effects in our analysis, we removed the ERY cluster and re-clustered prior to our analysis of actively cycling and proliferating cells. 8 principal components were selected for dimensionality reduction and combined with a resolution of 0.5, to visualize transcriptional heterogeneity across human development spanning 3 to 24 pcw. Cell cycle phase was determined with ‘CellCycleScoring’ to identify actively proliferating cells. Only ages with more than 50 cells were selected for the proliferation wave signature. Data were visualized using DimPlot, FeaturePlot, VlnPlot and DoHeatmap functions, and differential expression analysis was performed with MAST (Finak et al., 2015). Alignment of actively cycling and proliferating cells between source data was done utilizing the ‘FindConservedMarkers’ function of the Seurat package and gene ontology and protein-protein interaction enrichment analyses was performed with *Metascape*.

### Quantification and statistical analysis

Statistical analysis and visualisations were done in RStudio and GraphPad Prism. We have used non-parametric techniques in our analyses and therefore, made no assumptions about data distributions or homoscedasticity. All information about statistical details per experiment can be found in the figure captions and the results section. In brief here, non-parametric correlations using Spearman’s tested associations between brain weight, layer thicknesses, age and densities with different markers. Sex differences were assessed using cumulative distribution plots with the 2-sample Kolmogorov-Smirnov test and means’ differences were tested using the Mann-Witney U test. Friedman’s test was used to test for differences between layers within matched data. Wilcoxon test was used to test differences between matched data (liver and brain from the same cases for example). We also fitted non-parametric regression lines to density and proliferation data using the following parameters: a Loess regression function with a medium number of 10 points followed by a smoothing spline function with 6-8 knots as smoothing factors, which were both recommended by Graphpad Prism. We tested centred polynomial models of up to the maximum recommended order (6^th^ order) to model our data but these could explain at most 60% of the variance. Therefore, these were not suitable and we opted for presenting all datapoints instead and applying non-parametric functions that follow the data trend with no assumptions about a model that could fit all data. Increasing the order of polynomial models may have improved variance but would have resulted in overfitting. Furthermore, biological fluctuations that we report in the microglial population underlie the difficulty of finding one model that fits all data. To test multimodality of data distributions, we used a non-parametric bootstrap approach with B=100 replicas and (Ameijeiras-Alonso et al., 2019) excess test which if significant would accept the null-hypothesis that our data distribution for proliferation or density had a number of modes that was greater than 1. This test was run on the entire dataset (3 pcw – 75 years of age, n=95) and we have reported the modes and the antimodes. To test for significance between temporal windows for density and proliferation, we compared mean ranks in temporal windows around the fitted regression lines using a non-parametric Kruskal-Wallis test corrected for multiple comparisons using Graphpad’s recommended tests (Dunn’s, Benjamini-Krieger) and report throughout the adjusted p-values. Temporal windows were either equally-spaced and data were grouped accordingly. To place these temporal windows within relevant human milestones, we also grouped data according to existing nomenclature as follows: embryonic (3-8 pcw), early fetal (9-15 pcw), mid-late fetal (16-25 pcw), preterm (26-35 pcw), term (36 pcw-birth), neonatal (0-1 month), infant (1 month-12 months), child (1 year-2 years, only in our study because we did not have samples between 2-12 years), and adult (>18 years) (Silbereis et al., 2016; Kostović et al., 2002; Carroll et al., 2021). For postnatal stages, we used the Mann-Whitney U non-parametric test to report significant differences between means. For RNA-seq data (single cell and bulk), p-values, including those related to analysis of DE genes, were corrected for FDR in EdgeR using the Benjamini-Hochberg correction. Volcano plots and heatmaps were plotted in RStudio. Hierarchical clustering was carried out in Trinity (Haas et al., 2013). Histological heatmaps were plotted using QGIS spatial analysis software as elaborated upon in the relevant STAR Methods section.

## Supplemental information

Document S1. Figures S1–S6 and Tables S1, S2, and S4

Table S3. Maternal demographics, related to Figures 1, 2, 3, and 4 and STAR Methods

Data S1. RNAseq gene lists, related to Figures 1 and 5 and STAR Methods

Data S2. Gene lists used for scRNAseq analysis, related to Figure 5 and STAR Methods

Document S2. Article plus supplemental information

## References

[bib1] Ameijeiras-Alonso J., Crujeiras R.M., Rodríguez-Casal A. (2019). Mode testing, critical bandwidth and excess mass. Test.

[bib2] Anders S., Pyl P.T., Huber W. (2015). HTSeq—a Python framework to work with high-throughput sequencing data. Bioinformatics.

[bib3] Askew K., Li K., Olmos-Alonso A., Garcia-Moreno F., Liang Y., Richardson P., Tipton T., Chapman M.A., Riecken K., Beccari S. (2017). Coupled proliferation and apoptosis maintain the rapid turnover of microglia in the adult brain. Cell Rep..

[bib4] Badimon A., Strasburger H.J., Ayata P., Chen X., Nair A., Ikegami A., Hwang P., Chan A.T., Graves S.M., Uweru J.O. (2020). Negative feedback control of neuronal activity by microglia. Nature.

[bib5] Bayer S.A., Altman J. (2006).

[bib6] Bayer S.A., Altman J. (2008).

[bib7] Bennett F.C., Bennett M.L., Yaqoob F., Mulinyawe S.B., Grant G.A., Hayden Gephart M., Plowey E.D., Barres B.A. (2018). A combination of ontogeny and CNS environment establishes microglial identity. Neuron.

[bib8] Bennett M.L., Bennett F.C., Liddelow S.A., Ajami B., Zamanian J.L., Fernhoff N.B., Mulinyawe S.B., Bohlen C.J., Adil A., Tucker A. (2016). New tools for studying microglia in the mouse and human CNS. Proc. Natl. Acad. Sci. USA.

[bib9] Bian Z., Gong Y., Huang T., Lee C.Z.W., Bian L., Bai Z., Shi H., Zeng Y., Liu C., He J. (2020). Deciphering human macrophage development at single-cell resolution. Nature.

[bib10] Cao J., O’Day D.R., Pliner H.A., Kingsley P.D., Deng M., Daza R.M., Zager M.A., Aldinger K.A., Blecher-Gonen R., Zhang F. (2020). A human cell atlas of fetal gene expression. Science.

[bib11] Carroll L., Braeutigam S., Dawes J.M., Krsnik Z., Kostovic I., Coutinho E., Dewing J.M., Horton C.A., Gomez-Nicola D., Menassa D.A. (2021). Autism spectrum disorders: multiple routes to, and multiple consequences of, abnormal synaptic function and connectivity. Neuroscientist.

[bib12] Coutinho E., Menassa D.A., Jacobson L., West S.J., Domingos J., Moloney T.C., Lang B., Harrison P.J., Bennett D.L.H., Bannerman D., Vincent A. (2017). Persistent microglial activation and synaptic loss with behavioral abnormalities in mouse offspring exposed to CASPR2-antibodies in utero. Acta Neuropathol..

[bib13] Cuadros M.A., Navascués J. (1998). The origin and differentiation of microglial cells during development. Prog. Neurobiol..

[bib14] Cunningham C.L., Martínez-Cerdeño V., Noctor S.C. (2013). Microglia regulate the number of neural precursor cells in the developing cerebral cortex. J. Neurosci..

[bib15] Daniszewski M., Senabouth A., Nguyen Q.H., Crombie D.E., Lukowski S.W., Kulkarni T., Sluch V.M., Jabbari J.S., Chamling X., Zack D.J. (2018). Single cell RNA sequencing of stem cell-derived retinal ganglion cells. Sci. Data.

[bib16] Deoni S.C., Dean D.C., Remer J., Dirks H., O’Muircheartaigh J. (2015). Cortical maturation and myelination in healthy toddlers and young children. Neuroimage.

[bib17] Dobin A., Davis C.A., Schlesinger F., Drenkow J., Zaleski C., Jha S., Batut P., Chaisson M., Gingeras T.R. (2013). STAR: ultrafast universal RNA-seq aligner. Bioinformatics.

[bib18] Duque A., Krsnik Z., Kostović I., Rakic P. (2016). Secondary expansion of the transient subplate zone in the developing cerebrum of human and nonhuman primates. Proc. Natl. Acad. Sci. USA.

[bib19] Engle W.A., American Academy of Pediatrics Committee on Fetus and Newborn (2004). Age terminology during the perinatal period. Pediatrics.

[bib20] Fan X., Fu Y., Zhou X., Sun L., Yang M., Wang M., Chen R., Wu Q., Yong J., Dong J. (2020). Single-cell transcriptome analysis reveals cell lineage specification in temporal-spatial patterns in human cortical development. Sci. Adv..

[bib21] Finak G., Mcdavid A., Yajima M., Deng J., Gersuk V., Shalek A.K., Slichter C.K., Miller H.W., Mcelrath M.J., Prlic M. (2015). MAST: a flexible statistical framework for assessing transcriptional changes and characterizing heterogeneity in single-cell RNA sequencing data. Genome Biol..

[bib22] Galatro T.F., Holtman I.R., Lerario A.M., Vainchtein I.D., Brouwer N., Sola P.R., Veras M.M., Pereira T.F., Leite R.E.P., Möller T. (2017). Transcriptomic analysis of purified human cortical microglia reveals age-associated changes. Nat. Neurosci..

[bib23] Gallyas F. (1979). Silver staining of myelin by means of physical development. Neurol. Res..

[bib24] Gerrelli D., Lisgo S., Copp A.J., Lindsay S. (2015). Enabling research with human embryonic and fetal tissue resources. Development.

[bib25] Ginhoux F., Greter M., Leboeuf M., Nandi S., See P., Gokhan S., Mehler M.F., Conway S.J., Ng L.G., Stanley E.R. (2010). Fate mapping analysis reveals that adult microglia derive from primitive macrophages. Science.

[bib26] Gomez-Nicola D., Perry V.H. (2015). Microglial dynamics and role in the healthy and diseased brain: a paradigm of functional plasticity. Neuroscientist.

[bib27] Gosselin D., Link V.M., Romanoski C.E., Fonseca G.J., Eichenfield D.Z., Spann N.J., Stender J.D., Chun H.B., Garner H., Geissmann F., Glass C.K. (2014). Environment drives selection and function of enhancers controlling tissue-specific macrophage identities. Cell.

[bib28] Gosselin D., Skola D., Coufal N.G., Holtman I.R., Schlachetzki J.C.M., Sajti E., Jaeger B.N., O'Connor C., Fitzpatrick C., Pasillas M.P. (2017). An environment-dependent transcriptional network specifies human microglia identity. Science.

[bib29] Haas B.J., Papanicolaou A., Yassour M., Grabherr M., Blood P.D., Bowden J., Couger M.B., Eccles D., Li B., Lieber M. (2013). De novo transcript sequence reconstruction from RNA-seq using the Trinity platform for reference generation and analysis. Nat. Protoc..

[bib30] Hanamsagar R., Alter M.D., Block C.S., Sullivan H., Bolton J.L., Bilbo S.D. (2017). Generation of a microglial developmental index in mice and in humans reveals a sex difference in maturation and immune reactivity. Glia.

[bib31] Harry G.J. (2013). Microglia during development and aging. Pharmacol. Ther..

[bib32] Haymaker W., Adams R.D. (1982).

[bib33] Herculano-Houzel S., Von Bartheld C.S., Miller D.J., Kaas J.H. (2015). How to count cells: the advantages and disadvantages of the isotropic fractionator compared with stereology. Cell Tissue Res..

[bib34] Hrabač P., Bosak A., Vukšić M., Judaš M., Kostović I., Krsnik Ž. (2018). The Zagreb collection of human brains: entering the virtual world. Croat. Med. J..

[bib35] Janossy G., Bofill M., Poulter L.W., Rawlings E., Burford G.D., Navarrete C., Ziegler A., Kelemen E. (1986). Separate ontogeny of two macrophage-like accessory cell populations in the human fetus. J. Immunol..

[bib36] Kostović I. (2020). The enigmatic fetal subplate compartment forms an early tangential cortical nexus and provides the framework for construction of cortical connectivity. Prog. Neurobiol..

[bib37] Kostović I., Judas M., Rados M., Hrabac P. (2002). Laminar organization of the human fetal cerebrum revealed by histochemical markers and magnetic resonance imaging. Cereb. Cortex.

[bib38] Kracht L., Borggrewe M., Eskandar S., Brouwer N., Chuva De Sousa Lopes S.M., Laman J.D., Scherjon S.A., Prins J.R., Kooistra S.M., Eggen B.J.L. (2020). Human fetal microglia acquire homeostatic immune-sensing properties early in development. Science.

[bib39] Lavin Y., Winter D., Blecher-Gonen R., David E., Keren-Shaul H., Merad M., Jung S., Amit I. (2014). Tissue-resident macrophage enhancer landscapes are shaped by the local microenvironment. Cell.

[bib40] Li H., Handsaker B., Wysoker A., Fennell T., Ruan J., Homer N., Marth G., Abecasis G., Durbin R., 1000 Genome Project Data Processing Subgroup (2009). The Sequence Alignment/Map format and SAMtools. Bioinformatics.

[bib41] Li M., Santpere G., Imamura Kawasawa Y., Evgrafov O.V., Gulden F.O., Pochareddy S., Sunkin S.M., Li Z., Shin Y., Zhu Y. (2018). Integrative functional genomic analysis of human brain development and neuropsychiatric risks. Science.

[bib42] Lindsay S.J., Xu Y., Lisgo S.N., Harkin L.F., Copp A.J., Gerrelli D., Clowry G.J., Talbot A., Keogh M.J., Coxhead J. (2016). HDBR expression: a unique resource for global and individual gene expression studies during early human brain development. Front. Neuroanat..

[bib43] Love S.E., Perry A.E., Ironside J.W.E., Budka H.E., Greenfield J.G. (2015).

[bib44] Marín-Teva J.L., Almendros A., Calvente R., Cuadros M.A., Navascués J. (1998). Tangential migration of ameboid microglia in the developing quail retina: mechanism of migration and migratory behavior. Glia.

[bib45] Marín-Teva J.L., Calvente R., Cuadros M.A., Almendros A., Navascués J. (1999). Circumferential migration of ameboid microglia in the margin of the developing quail retina. Glia.

[bib46] Martín-Estebané M., Navascués J., Sierra-Martín A., Martín-Guerrero S.M., Cuadros M.A., Carrasco M.C., Marín-Teva J.L. (2017). Onset of microglial entry into developing quail retina coincides with increased expression of active caspase-3 and is mediated by extracellular ATP and UDP. PLoS One.

[bib47] Menassa D.A., Gomez-Nicola D. (2018). Microglial dynamics during human brain development. Front. Immunol..

[bib48] Mittelbronn M., Dietz K., Schluesener H.J., Meyermann R. (2001). Local distribution of microglia in the normal adult human central nervous system differs by up to one order of magnitude. Acta Neuropathol..

[bib49] Monier A., Adle-Biassette H., Delezoide A.L., Evrard P., Gressens P., Verney C. (2007). Entry and distribution of microglial cells in human embryonic and fetal cerebral cortex. J. Neuropathol. Exp. Neurol..

[bib50] Monier A., Evrard P., Gressens P., Verney C. (2006). Distribution and differentiation of microglia in the human encephalon during the first two trimesters of gestation. J. Comp. Neurol..

[bib51] Nikodemova M., Kimyon R.S., De I., Small A.L., Collier L.S., Watters J.J. (2015). Microglial numbers attain adult levels after undergoing a rapid decrease in cell number in the third postnatal week. J. Neuroimmunol..

[bib52] Nogales F.F. (1993).

[bib53] Olmos-Alonso A., Schetters S.T., Sri S., Askew K., Mancuso R., Vargas-Caballero M., Holscher C., Perry V.H., Gomez-Nicola D. (2016). Pharmacological targeting of CSF1R inhibits microglial proliferation and prevents the progression of Alzheimer’s-like pathology. Brain.

[bib54] O’Rahilly R., Müller F. (2010). Developmental stages in human embryos: revised and new measurements. Cells Tissues Organs.

[bib55] Paolicelli R.C., Bolasco G., Pagani F., Maggi L., Scianni M., Panzanelli P., Giustetto M., Ferreira T.A., Guiducci E., Dumas L. (2011). Synaptic pruning by microglia is necessary for normal brain development. Science.

[bib56] Park J.E., Jardine L., Gottgens B., Teichmann S.A., Haniffa M. (2020). Prenatal development of human immunity. Science.

[bib57] Penzes P., Cahill M.E., Jones K.A., Vanleeuwen J.E., Woolfrey K.M. (2011). Dendritic spine pathology in neuropsychiatric disorders. Nat. Neurosci..

[bib58] Pont-Lezica L., Beumer W., Colasse S., Drexhage H., Versnel M., Bessis A. (2014). Microglia shape corpus callosum axon tract fasciculation: functional impact of prenatal inflammation. Eur. J. Neurosci..

[bib59] Popescu D.M., Botting R.A., Stephenson E., Green K., Webb S., Jardine L., Calderbank E.F., Polanski K., Goh I., Efremova M. (2019). Decoding human fetal liver haematopoiesis. Nature.

[bib60] Réu P., Khosravi A., Bernard S., Mold J.E., Salehpour M., Alkass K., Perl S., Tisdale J., Possnert G., Druid H. (2017). The lifespan and turnover of microglia in the human brain. Cell Rep..

[bib61] Rezaie P., Male D. (1999). Colonisation of the developing human brain and spinal cord by microglia: a review. Microsc. Res. Tech..

[bib62] Robinson M.D., Mccarthy D.J., Smyth G.K. (2010). edgeR: a Bioconductor package for differential expression analysis of digital gene expression data. Bioinformatics.

[bib63] Sánchez-López A., Cuadros M.A., Calvente R., Tassi M., Marín-Teva J.L., Navascués J. (2004). Radial migration of developing microglial cells in quail retina: a confocal microscopy study. Glia.

[bib64] Schindelin J., Arganda-Carreras I., Frise E., Kaynig V., Longair M., Pietzsch T., Preibisch S., Rueden C., Saalfeld S., Schmid B. (2012). Fiji: an open-source platform for biological-image analysis. Nat. Methods.

[bib65] Schwarz J.M., Sholar P.W., Bilbo S.D. (2012). Sex differences in microglial colonization of the developing rat brain. J. Neurochem..

[bib66] Sekar A., Bialas A.R., de Rivera H., Davis A., Hammond T.R., Kamitaki N., Tooley K., Presumey J., Baum M., Van Doren V. (2016). Schizophrenia risk from complex variation of complement component 4. Nature.

[bib67] Sierra A., Abiega O., Shahraz A., Neumann H. (2013). Janus-faced microglia: beneficial and detrimental consequences of microglial phagocytosis. Front. Cell. Neurosci..

[bib68] Sierra A., Encinas J.M., Deudero J.J., Chancey J.H., Enikolopov G., Overstreet-Wadiche L.S., Tsirka S.E., Maletic-Savatic M. (2010). Microglia shape adult hippocampal neurogenesis through apoptosis-coupled phagocytosis. Cell Stem Cell.

[bib69] Silbereis J.C., Pochareddy S., Zhu Y., Li M., Sestan N. (2016). The cellular and molecular landscapes of the developing human central nervous system. Neuron.

[bib70] Soneson C., Robinson M.D. (2018). Bias, robustness and scalability in single-cell differential expression analysis. Nat. Methods.

[bib71] Squarzoni P., Oller G., Hoeffel G., Pont-Lezica L., Rostaing P., Low D., Bessis A., Ginhoux F., Garel S. (2014). Microglia modulate wiring of the embryonic forebrain. Cell Rep..

[bib72] Stuart T., Butler A., Hoffman P., Hafemeister C., Papalexi E., Mauck W.M., Hao Y., Stoeckius M., Smibert P., Satija R. (2019). Comprehensive integration of single-cell data. Cell.

[bib73] Swinnen N., Smolders S., Avila A., Notelaers K., Paesen R., Ameloot M., Brône B., Legendre P., Rigo J.M. (2013). Complex invasion pattern of the cerebral cortex bymicroglial cells during development of the mouse embryo. Glia.

[bib74] Tavian M., Péault B. (2005). Embryonic development of the human hematopoietic system. Int. J. Dev. Biol..

[bib75] Tetreault N.A., Hakeem A.Y., Jiang S., Williams B.A., Allman E., Wold B.J., Allman J.M. (2012). Microglia in the cerebral cortex in autism. J. Autism Dev. Disord..

[bib76] Vankriekelsvenne E., Chrzanowski U., Manzhula K., Greiner T., Wree A., Hawlitschka A., Llovera G., Zhan J., Joost S., Schmitz C. (2022). Transmembrane protein 119 is neither a specific nor a reliable marker for microglia. Glia.

[bib77] Velmeshev D., Schirmer L., Jung D., Haeussler M., Perez Y., Mayer S., Bhaduri A., Goyal N., Rowitch D.H., Kriegstein A.R. (2019). Single-cell genomics identifies cell type-specific molecular changes in autism. Science.

[bib78] Verney C., Monier A., Fallet-Bianco C., Gressens P. (2010). Early microglial colonization of the human forebrain and possible involvement in periventricular white-matter injury of preterm infants. J. Anat..

[bib79] Waise S., Parker R., Rose-Zerilli M.J.J., Layfield D.M., Wood O., West J., Ottensmeier C.H., Thomas G.J., Hanley C.J. (2019). An optimised tissue disaggregation and data processing pipeline for characterising fibroblast phenotypes using single-cell RNA sequencing. Sci. Rep..

