# Supplementary material for: The spatiotemporal dynamics of microglia across the human lifespan
Source: Dev Cell. 2022 Sep 12;57(17):2127–2139.e6. doi: 10.1016/j.devcel.2022.07.015 (PMC9616795; doi:10.1016/j.devcel.2022.07.015)
Supplement: Document S1. Figures S1–S6 and Tables S1, S2, and S4 [file mmc1.pdf]

Developmental Cell, Volume *57*

Supplemental information

The spatiotemporal dynamics of microglia across the human lifespan

David A. Menassa, Tim A.O. Muntslag, Maria Martin-Estebané, Liam Barry-Carroll, Mark A. Chapman, Istvan Adorjan, Teadora Tyler, Bethany Turnbull, Matthew J.J. Rose-Zerilli, James A.R. Nicoll, Zeljka Krsnik, Ivica Kostovic, and Diego Gomez-Nicola

SUPPLEMENTAL ITEMS

Index:

Figures S1 to S6

Tables S1, S2 and S4

SUPPLEMENTAL FIGURES

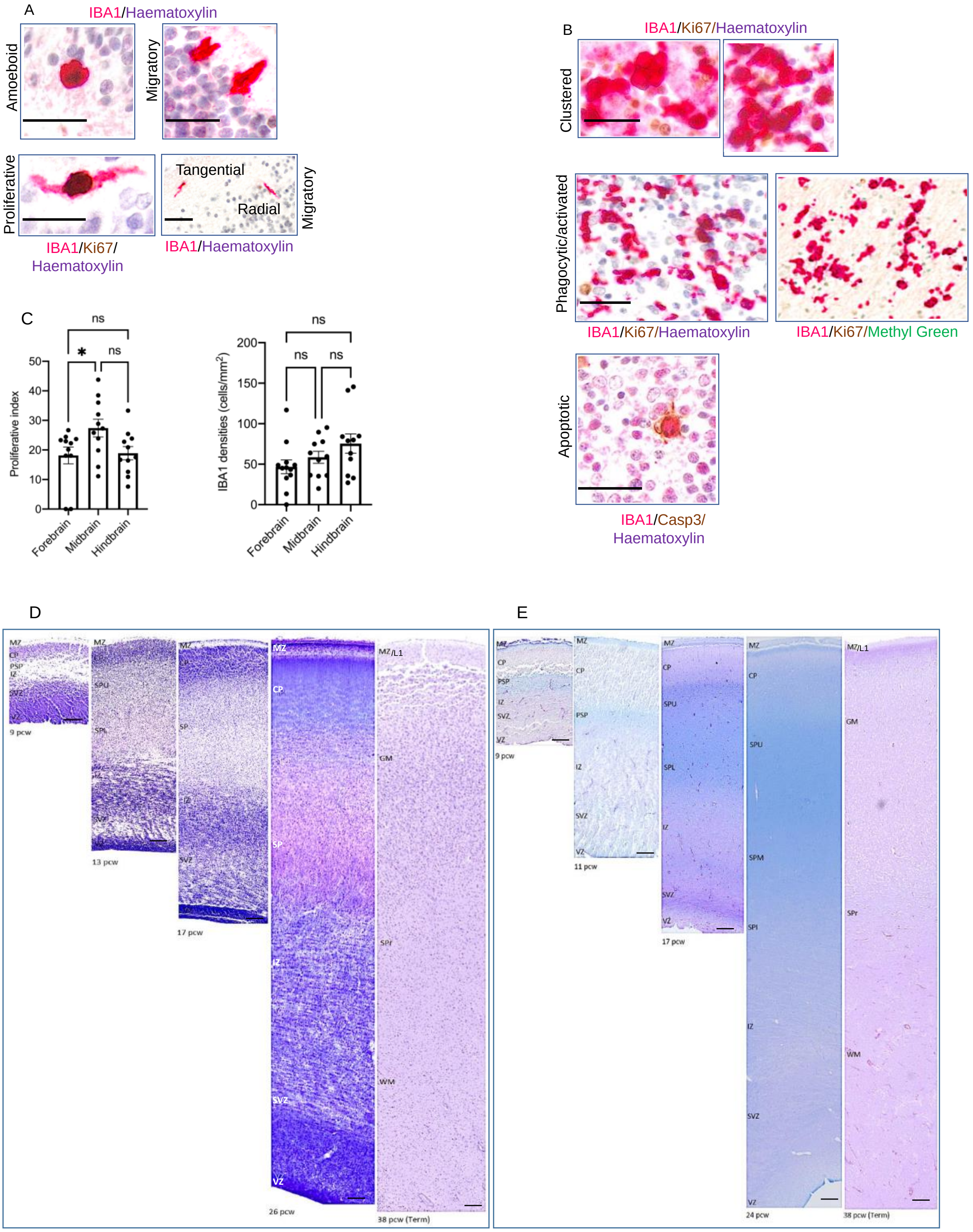

Figure S1, ***Related to figures 2, 3 & 4.*** (A, B) Microglia morphologies during development. (A) Microglial morphologies are amoeboid, proliferative, or migratory in the embryonic age (3-8 pcw) and (B), additional morphologies throughout fetal life (9-38 pcw) are seen such as clustered, phagocytic, and apoptotic. Scale bar: 50 µm. (C) Embryonic regional brain

proliferation and densities. Proliferative indices and IBA1+ densities between brain regions (n=14). **p<0.01, ns p>0.05*. All data are shown as mean $\pm$ SEM. (D, E) Delineation of anatomical boundaries in the developing cortex between 9 and 38 postconceptional weeks. (D) Nissl histochemistry of transient cortical zones. (E) Periodic-Acid Schiff/Alcian Blue histochemistry for marking the subplate in transient cortical zones. *CP*: cortical plate; *GM:* Grey matter; *IZ*: intermediate zone; *MZ:* marginal zone; *PSP*: presubplate; *pcw*: postconceptional week; *SP*: subplate; *SPU:* subplate upper portion; *SPM*: subplate middle portion; *SPI:* subplate inferior portion; *SPL*: subplate lower portion; *SPr*: subplate remnant; *SVZ*: subventricular zone; *VZ*: ventricular zone; *WM:* white matter. Scale bar: 100µm.

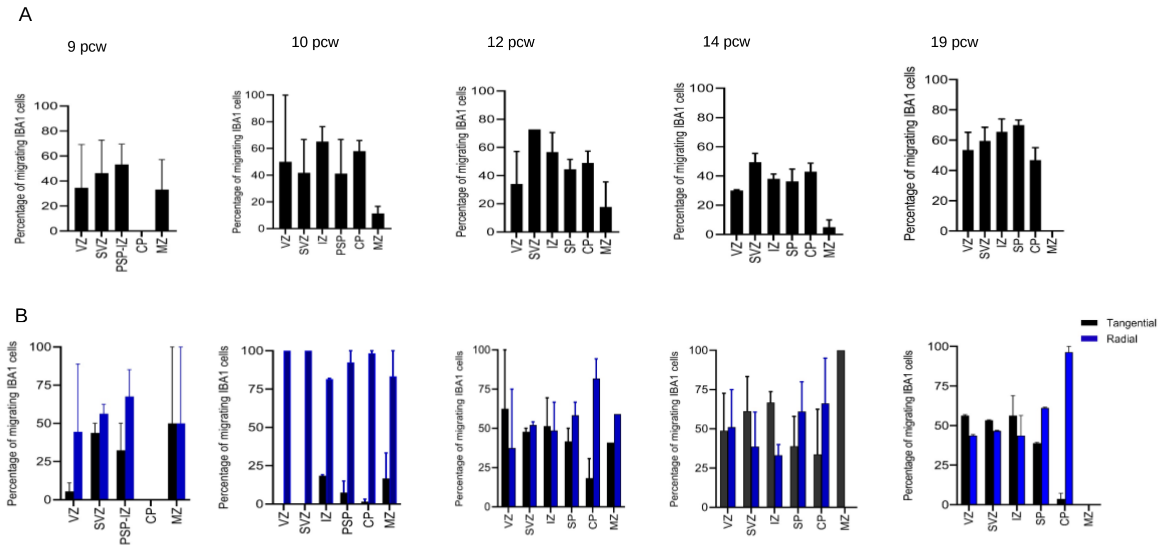

Figure S2. Migratory microglial profile and phenotype during development, *Related to figures 2 & 3.*

(A) Percentage of IBA1 cells migrating into the various transient zones (n=2 for each timepoint). At 9 pcw, the CP has no migrating microglia whilst most layers have substantial numbers of migrating cells. From 10-14 pcw, migrating cells are found in all layers and by 19 pcw, migrating cells can no longer be seen present in the MZ. (B) Type of migration in each transient layer (n=2 for each timepoint). Migration is radial and/or tangential depending on each layer across 9-19 pcw. *CP :* cortical plate ; *IZ :* intermediate zone ; *MZ :* marginal zone ; *PSP* : presubplate; *pcw*: postconceptional week; *SP*: subplate;; *SVZ*: subventricular zone; *VZ*: ventricular zone.

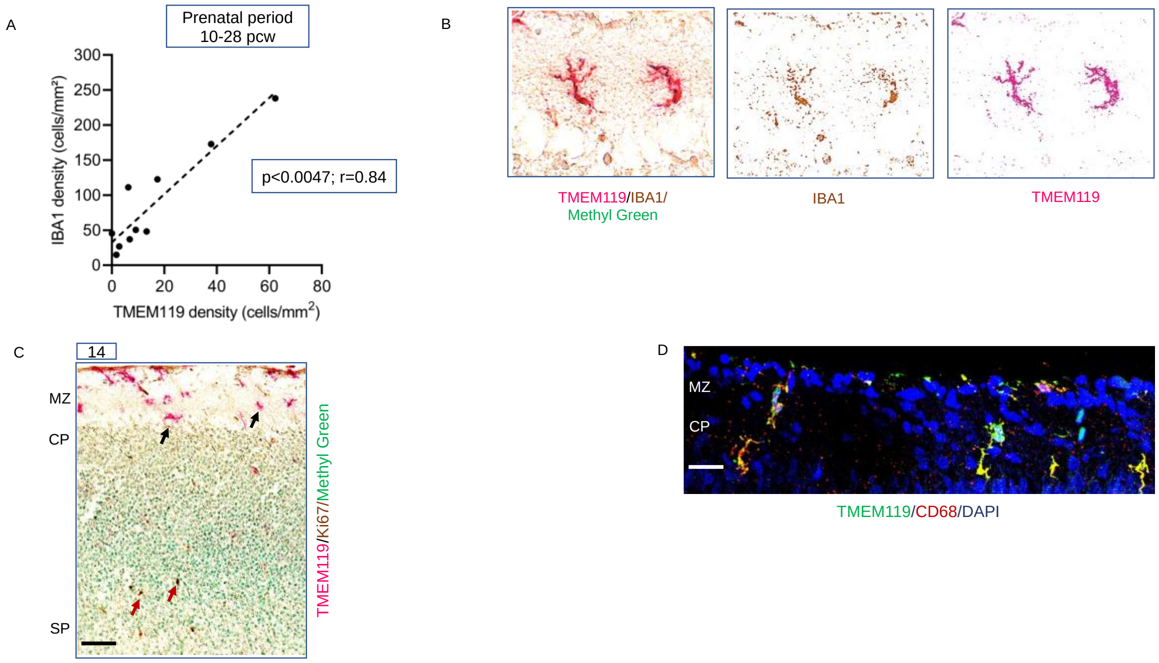

Figure S3. TMEM119 expression during prenatal development, *Related to figure 3*. (A) IBA1 densities correlative positively with TMEM119 densities (n=10 cases between 10-28 pcw) (r=0.84, p<0.0047). (B) Deconvolution of signal showing that TMEM119 and IBA1 colocalise. (C) TMEM119^{+} cells are not proliferative in the MZ (black arrows) in a representative 14 pcw neocortical column. Proliferation (non-microglial) is shown in the deeper layers (red arrows for the Ki67 signal). Scale bar: 100 µm. (D) TMEM119^{+} cells co-express CD68 lysosomal marker in the MZ. Scale bar: 50 µm. *CP*: cortical plate; *MZ*: marginal zone; *SP*: subplate.

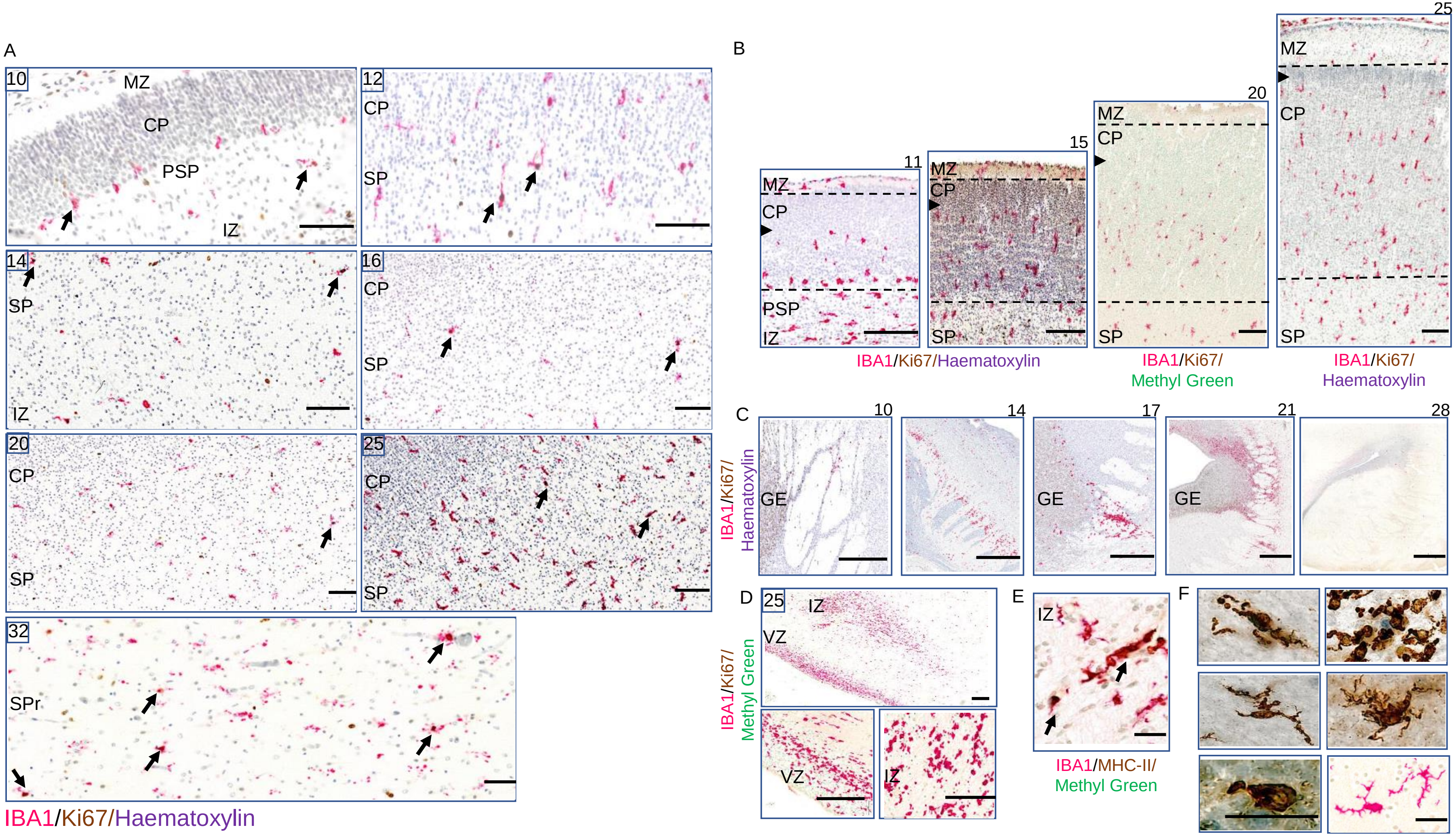
A
10
MZ
CP
PSP
IZ
12
CP
SP
14
SP
IZ
16
CP
SP
20
CP
SP
25
CP
SP
32
SPr
IBA1/Ki67/Haematoxylin
B
11
MZ
CP
PSP
IZ
15
MZ
CP
SP
20
MZ
CP
SP
25
MZ
CP
SP
IBA1/Ki67/Haematoxylin
IBA1/Ki67/
Methyl Green
IBA1/Ki67/
Haematoxylin
C
IBA1/Ki67/
Haematoxylin
10
14
17
21
28
GE
GE
GE
D
IBA1/Ki67/
Methyl Green
25
IZ
VZ
VZ
IZ
E
IZ
IBA1/MHC-II/
Methyl Green
F

Figure S4. Descriptive account of regional findings across development, *Related to figures 2, 3 & 4.*

(A) Proliferation of microglial cells is highest in the subplate region across gestation. Scale bar: 100 µm (B) Microglial cells invade the upper portion of the cortical plate by 25 pcw and spare it before that time. Scale bar: 100 µm (C) Ventral telencephalon microglia cluster around the internal and external capsules. These clusters increase during gestation and disappear by 28 pcw. Scale bar: 500 µm (D) Microglial hotspots can be seen in the intermediate zone during most of gestation and resolve by the 32nd pcw. Scale bar: 100 µm (E) Microglia are positive for MHCII. Scale bar: 75 µm (E) and towards the late third trimester, cluster in the ventricular zone. (F) Range of microglial morphologies from amoeboid to intermediate and ramified mature types can be seen in transient layers developmentally. Scale bar: 75 µm. *CP :* cortical plate; *GE:* ganglionic eminence; *IZ :* intermediate zone ; *MZ :* marginal zone ; *PSP* : presubplate; *pcw*: postconceptional week; *SP*: subplate;; *SVZ*: subventricular zone; *VZ*: ventricular zone.

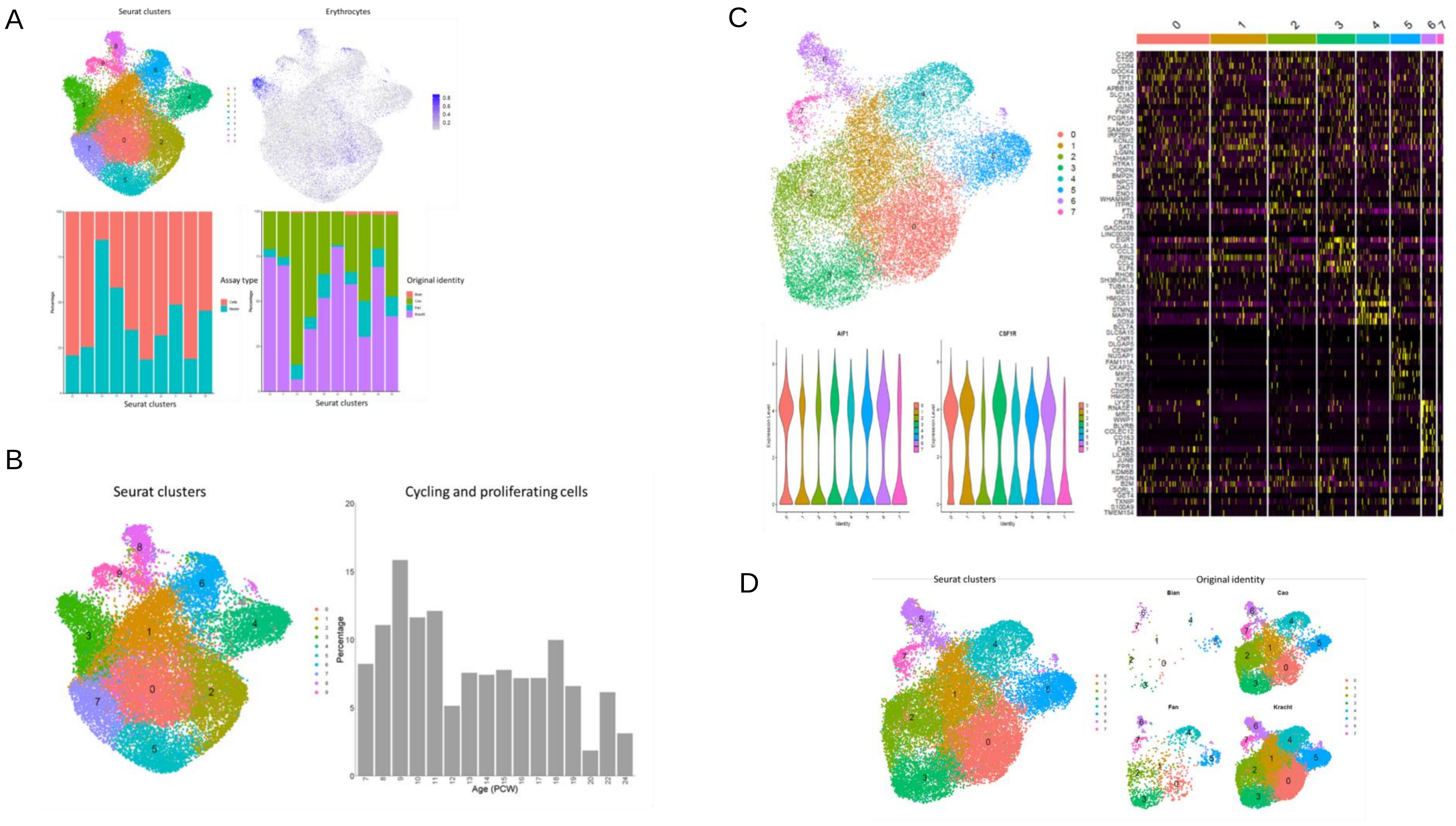
A
Seurat clusters
Erythrocytes
Assay type
Original identity
Seurat clusters
Seurat clusters
B
Seurat clusters
Cycling and proliferating cells
Percentage
Age (PCW)
C
AIF1
CSF1R
D
Seurat clusters
Original identity
Bian
Cao
Fan
Kracht

Figure S5. Single-cell RNA-seq analysis, *Related to figure 5*. (A) Preliminary analysis identified a cluster enriched for erythrocyte (ERY) markers (e.g. HBG2, HBB). The single-nuclei dataset by (Cao et al. 2020) contributed most cells of this ERY cluster, suggesting that this cluster is a technical, method-specific artefact, albeit all datasets showed some degree of ERY. We removed the ERY cluster and re-clustered prior to our analysis of cycling proliferating cells. (B) Seurat clusters before the removal of the ERY cluster (left). Cycling and proliferating cell pattern was not affected. (C) 8 transcriptionally distinct clusters were identified in our integrated object. The heatmap displays the top 5 DEG of each cluster, as identified with the FindAllMarkers function (MAST). Several developmental homeostatic microglia were identified as well as several previously identified microglial subtypes (CPM, CAM, BAM). Of note, one cluster displayed markers not commonly associated with microglia with NPC-linked genes. which may suggest impurities in preparation; or capture a novel microglial subtype that restricts the progenitor pool. (D) Spatial distribution of gestational myeloid cells by original identity. *CS*: Carnegie stage; *GW/GA*: gestational week/age; *PCD:* postconceptional day; *PCW*: postconceptional week.

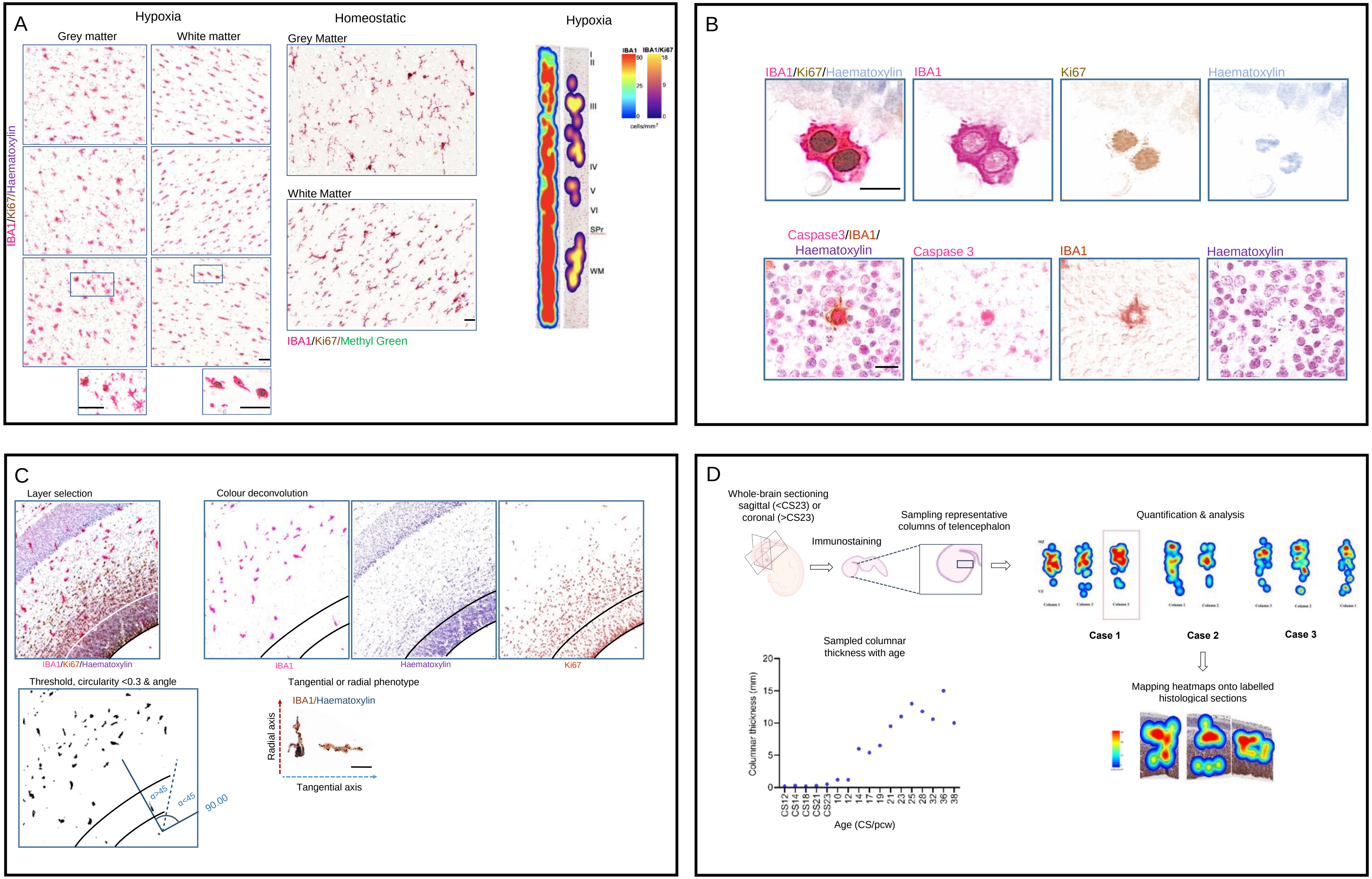
A
Hypoxia
Grey matter
White matter
Homeostatic
Grey Matter
White Matter
IBA1/Ki67/Methyl Green
IBA1/Ki67/Haematoxylin
Hypoxia
I
II
III
IV
V
VI
SPr
WM
B
IBA1/Ki67/Haematoxylin
IBA1
Ki67
Haematoxylin
Caspase3/IBA1/
Haematoxylin
Caspase 3
IBA1
Haematoxylin
C
Layer selection
Colour deconvolution
IBA1/Ki67/Haematoxylin
IBA1
Haematoxylin
Ki67
Threshold, circularity <0.3 & angle
α>45
α<45
90.00
Tangential or radial phenotype
IBA1/Haematoxylin
Radial axis
Tangential axis
D
Whole-brain sectioning
sagittal (<CS23) or
coronal (>CS23)
Immunostaining
Sampling representative
columns of telencephalon
Quantification & analysis
Case 1
Case 2
Case 3
Sampled columnar
thickness with age
Columnar thickness (mm)
Age (CS/pcw)
Mapping heatmaps onto labelled
histological sections

Figure S6. Tissue quality control and analysis, Related to Methods and Figures 1 & 4. (A) Reaction of microglia to hypoxia. Representative photomicrographs of microglia reactive to hypoxic-ischaemic injury in a 38 postconceptional term grey and white matters (Fetus 52 in table 7) and homeostatic microglial ramified morphology with a lower proliferation rate in the absence of hypoxic injury in a 38 postconceptional week case included in our study Note the amoeboid shape and higher proliferation rate compared to homeostatic microglia. Representative heatmap of proliferation and density along the cortical column. *SPr*: subplate remnant; *WM:* white matter. Scale bar: 50μm. (B) Deconvolution of signal in brightfield. Example of colour deconvolution in brightfield for proliferative microglia (IBA1/Ki67/Haematoxylin). Example of colour deconvolution in brightfield for cell death (Caspase 3/IBA1/Haematoxylin). Scale bar: 10μm.(C) Migration analysis workflow: Layer selection, Deconvolution and Thresholded photomicrograph with particle selection according to a circularity criterion <0.3 for a migratory cell phenotype. The angle is recorded between the major axis of the cell and the layer plane with α<45 for tangential and α>45 for radial migrations. Tangential and radial microglial phenotypes. Scale bar: 30μm. Figure S12. Heatmap analysis, Related to figure 4 & methods. (D) Workflow of image processing to obtain heatmaps from cortical columns. Columnar thickness measurements for heatmaps against age. CS: Carnegie stages.

Table S1. Data source characteristics, *Related to figure 5 & methods.*

	Source				
**Age (pcw)**	*Bian et al. (2020)*	*Cao et al. (2020)*	*Fan et al. (2020)*	*Kracht et al. (2020)*	**# (of cells)**
3	9				9
4	32				32
5	24		1		25
6	7		28		35
7			17	2872	2889
8	63		28	1926	2017
9			11	2467	2478
10			73	1806	1879
11			31	767	798
12			66		66
13		1726		2175	3901
14			64	876	940
15			303	842	1145
16		3032		842	3874
17		458			458
18		856	122		978
19			149		149
20			40		40
21					
22			193		193
23					
24			232		232

**pcw: postconceptional weeks*

**Table S2. Developmental demographics, *Related to figure 1, 2, 3, 4 & methods*.**							
**Case**	**GA (CS)**	**PCW**	**Sex**	**PMI (h)**	**BW (g)**	**Cause of Death**	**Histology**
Embryo	5 (10)	Late 3rd	F	3.67	n/a	Elective termination	Normal
Embryo	6 (12)	4	M	3	n/a	Elective termination	Normal
Embryo	6 (12)	4	F	3.92	n/a	Elective termination	Normal
Embryo	6 (12)	4	F	4.67	n/a	Elective termination	Normal
Embryo	7 (14)	5	M	7	n/a	Elective termination	Normal
Embryo	7 (14)	5	F	6.42	n/a	Elective termination	Normal
Embryo	7 (14)	5	M	n/k	n/a	Elective termination	Normal
Embryo	8 (16)	6	M	2.75	n/a	Elective termination	Normal
Embryo	8 (16)	6	F	2.5	n/a	Elective termination	Normal
Embryo	9 (18)	7	M	5.25	n/a	Elective termination	Normal
Embryo	9 (18)	7	F	n/k	n/a	Elective termination	Normal
Embryo	10 (21)	8	M	1.25	n/a	Elective termination	Normal
Embryo	10 (21)	8	F	n/k	n/a	Elective termination	Normal
Embryo	11 (23)	9	M	n/k	n/k	Elective termination	Normal
Embryo	11 (23)	9	F	16.67	n/k	Elective termination	Normal
Fetus	12	10	F	16.67	n/k	Elective termination	Normal
Fetus	12	10	M	16.67	n/k	Elective termination	Normal
Fetus	12	10	n/k	3.33	n/k	Elective termination	Normal
Fetus	13	11	M	2	n/k	Termination of pregnancy	Normal
Fetus	13	11	M	n/k	n/k	Elective termination	Normal
Fetus	14	12	F	2.5	n/k	Elective termination	Normal
Fetus	14	12	F	120	10	Elective termination	Normal
Fetus	14	12	F	16.67	n/k	Elective termination	Normal
Fetus	15	13	M	n/k	n/k	Termination of pregnancy	Normal
Fetus	16	14	F	16.00	n/k	Elective termination	Normal
Fetus	16	14	M	n/k	n/k	Termination of pregnancy	Normal
Fetus	17	15	M	n/k	n/k	Termination of pregnancy	Normal

Fetus	17	15	F	n/k	n/k	Elective termination	Normal
Fetus	18	16	M	n/k	33	Elective termination	Normal
Fetus	18	16	M	16.67	n/k	Elective termination	Normal
Fetus	19	17	F	144	41	Elective termination	Normal
Fetus	19	17	M	n/k	n/k	Miscarriage	Normal
Fetus	20	18	M	16.67	n/k	Elective termination	
Fetus	21	19	M	48	65	Termination of pregnancy	Normal
Fetus	21	19	M	24	67	Miscarriage	Normal
Fetus	22	20	M	72	93	Miscarriage	Normal
Fetus	23	21	F	n/k	n/k	Termination of pregnancy	Normal
Fetus	25	23	M	48	121	Termination of pregnancy	Normal
Fetus	25	23	M	n/k	110	Intrauterine death	Normal
Fetus	26	24	F	n/k	125	Preterm birth	Normal
Fetus	27	25	F	6	n/k	Intrauterine death	Normal
Fetus	27	25	F	n/k	n/k	Preterm birth, defective placenta	Normal
Fetus	28	26	M	96	159	n/k	Normal
Fetus	28	26	M	n/k	n/k	n/k	Normal
Fetus	30	28	F	240	n/k	Maternal road accident/placental separation	Normal frontal histology
Fetus	34	32	M	144	312	Respiratory distress syndrome	Normal
Fetus	35	33	F	n/k	295	Pulmonary hypoplasia, preterm birth	Normal
Fetus	38	36	F	17	380	Preterm birth	Normal
Fetus	40	38	M	5	n/k	Perinatal asphyxia, cardiorespiratory arrest	Normal
Fetus	40	38	F	5	n/k	Placental disruption, *in utero* death	Normal
Fetus	40	38	M	n/k	370	Septicaemia, premature birth	Normal
Fetus †	40	38	M	72	356	Intrauterine death	Hypoxic injury

Total number of cases shown is n = 52. In total, 63 cases were assessed. † Only one hypoxic case is included in the table above for reference. Refer also to supplementary figure 1 for a representative example of excluded cases due to hypoxic injury to the area. *BW*: brain weight; *CS:* Carnegie stage; *F:* female; *GA:* gestational age; *M:* male; *n/a:* not applicable; *n/k:* not known; *PCW*: postconceptional week; *PMI*: post-mortem interval.

Table S4. Postnatal demographics, *Related to figure 1, 6 & methods.*

Case	Age (years)	Sex	PMI (h)	BW (g)	Cause of Death	Histology
Neonate	0.003*	F	n/k	390	Intrapartum asphyxia	Normal
Neonate	0.008*	F	n/k	400	SUDI	Normal
Neonate	0.01*	M	n/k	415	Intrapartum asphyxia	Normal
Neonate	0.02*	M	n/k	375	n/k	Normal
Neonate	0.03*	M	n/k	350	SUDI	Normal
Neonate	0.03*	F	n/k	335	SUDI	Normal
Neonate	0.05*	M	n/k	n/k	Respiratory arrest	Normal
Neonate	0.07*	F	n/k	395	Intrapartum asphyxia	Normal
Neonate	0.07*	M	n/k	465	SUDI	Normal
Neonate	0.08*	F	n/k	465	SUDI	Normal
Neonate	0.08*	F	n/k	525	SUDI	Normal
Infant	0.14*	F	n/k	535	Pneumonia	Normal
Infant	0.17*	M	n/k	530	SUDI	Normal
Infant	0.17*	M	n/k	555	SUDI	Normal
Infant	0.25*	M	72	598	SUDI	Normal
Infant	0.33*	M	n/k	840	SUDI	Normal
Infant	0.42*	F	n/k	485	SUDI	Normal
Infant	0.5*	M	n/k	885	SUDI	Normal
Infant	0.50*	M	n/k	n/k	n/k	Normal
Infant	0.92*	F	96	1008	SUDEP	Normal
Infant	0.92*	M	96	1288	Subdural haemorrhage	Normal
Child	1	M	48	1342	SUDEP	Normal
Child	1.5	F	n/k	1295	Aspiration, SUDEP	Normal
Child	2	M	96	1379	SUDEP	Normal
Adult	18	M	5	n/k	Car crash	Normal
Adult	25	M	53	1640	Suspension by ligature	Normal
Adult	25	M	81	1500	Road traffic collision	Normal

Adult	27	M	67	1540	Ischaemic heart disease	Normal
Adult	30	M	71	1670	n/k	Normal
Adult	35	F	44	1240	n/k	Normal
Adult	37	F	46	1290	Hepatic failure	Normal
Adult	40	M	48	1320	Pulmonary embolism	Normal
Adult	42	M	103	1560	Suspension by ligature	Normal
Adult	51	M	51	1460	Coronary artery atheroma	Normal
Adult	56	M	44	1500	Ischaemic heart disease	Normal
Adult	53	M	64	1520	Depressive episode	Normal
Adult	57	F	73	1320	Sudden death	Normal
Adult	59	F	50	1500	Cardiomegaly	Normal
Adult	60	M	52	1460	Ischaemic heart disease	Normal
Adult	66	M	48	1350	Hypertensive heart disease	Normal
Adult	71	F	41	1210	Ischaemic heart disease	Normal
Adult	74	F	41	1520	Pulmonary embolism	Normal
Adult	74	M	23	1350	Ischaemic heart disease	Normal
Adult	74	M	12	1600	Pulmonary embolism	Normal
Adult	75	F	24	1580	Ischaemic heart disease	Normal

Total number of cases shown is n = 45, total cases assessed is 71 (Excluded cases due to trauma, malformation, hypoxic injury to the area or loss of antigenicity). *BW:* brain weight; *F:* female; *M:* male; *n/k:* not known; *PMI:* post-mortem interval; *SUDEP*: sudden unexpected death in epilepsy; *SUDI*: sudden unexpected death in infancy; **Age range in days for cases < 1 year*: (1 day - 335 days).
